# Inactivation of AXL in Cardiac Fibroblasts Alleviates Right Ventricular Remodeling in Pulmonary Hypertension

**DOI:** 10.1002/advs.202508995

**Published:** 2025-12-03

**Authors:** Li‐Wei Wu, Min Chen, Chen‐Yu Jiang, Dai‐Ji Jiang, Xu Zhang, Xiao‐He Xu, Yi‐Wei Liu, Bei Feng, Lin‐Cai Ye, Yang‐Yang He, Xu Huang, Yi‐Chi Zhang, Xing‐Liang Zhou, Yi Shen, Tian‐Yu Liu, Li‐Jun Fu, Yi Yan, Hao Zhang

**Affiliations:** ^1^ Heart Center and Shanghai Institute of Pediatric Congenital Heart Disease Shanghai Children's Medical Center National Children's Medical Center Shanghai Jiao Tong University School of Medicine Shanghai 200127 China; ^2^ Department of Cardiothoracic Surgery Shanghai Children's Medical Center National Children's Medical Center Shanghai Jiao Tong University School of Medicine Shanghai 200127 China; ^3^ Children's Heart Center Institute of Cardiovascular Development and Translational Medicine The Second Affiliated Hospital and Yuying Children's Hospital Wenzhou Medical University Wenzhou 325027 China; ^4^ School of Pharmacy Henan University Kaifeng 475004 China; ^5^ Shanghai Research Center for Pediatric Cardiovascular Diseases Shanghai Children's Medical Center National Children's Medical Center Shanghai Jiao Tong University School of Medicine Shanghai 200127 China; ^6^ Department of Cardiology Shanghai Children's Medical Center National Children's Medical Center Shanghai Jiao Tong University School of Medicine Shanghai 200127 China

**Keywords:** AXL, pulmonary hypertension, right ventricular remodeling, single‐nucleus RNA sequencing, transcription factor nuclear factor I C

## Abstract

Right ventricular (RV) adaptation critically determines survival in pulmonary hypertension (PH). Since cardiac fibroblasts (FBs) are crucial mediators of cardiac fibrosis, we aim to uncover the mechanism underlying their activation during RV remodeling. Using single‐nucleus RNA sequencing (snRNA‐seq) across three rodent PH models—hypobaric hypoxia and Sugen 5416/hypoxia in mice, and monocrotaline in rats—we identified elevated Axl expression in RV FBs under PH. AXL upregulation was consistently observed in RV FBs from both human PH patients and animal models. Cardiac FB‐specific Axl overexpression exacerbated RV remodeling in both PH and pulmonary artery banding (PAB) models, whereas Axl knockdown in FBs alleviated this remodeling. Functionally, AXL promoted FB proliferation, migration, and extracellular matrix synthesis via the PI3K‐AKT pathway, facilitating nuclear translocation of NFIC, which in turn promoted the transcription of targeted genes such as COL1A1. Inhibiting PI3K or administering R428 mitigated AXL‐driven RV remodeling in PH, and R428 also ameliorated remodeling in PAB mice. In conclusion, AXL signals the PI3K‐AKT pathway to license nuclear translocation of NFIC, thereby dictating the transcription of fibrotic genes in FBs and driving RV remodeling. These findings reveal novel insights into RV pathophysiology and highlight AXL as a potential therapeutic target for PH‐induced RV remodeling.

## Graphical abstract

Pulmonary hypertension (PH) is a progressive condition with high morbidity and mortality, largely owing to right ventricular (RV) failure resulting from maladaptive remodeling. Our study provides strong evidence in support of a critical, detrimental role for AXL as a previously unrecognized determinant driving RV fibrotic pathology in PH. AXL signals PI3K/AKT pathway to license nuclear translocation of NFIC, thereby dictating the transcription of targeted genes in support of FB activation (Created in BioRender. Yan, Y. (2025) https://BioRender.com/s34e956).

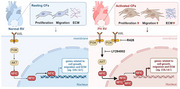

## 1 Introduction

Pulmonary hypertension (PH) is a progressive disorder characterized by elevated pulmonary vascular resistance due to various causes.^[^ ^1^, ^2^, ^3^ ^]^ It often leads to right ventricular (RV) remodeling and dysfunction, which can eventually result in death. Despite advances in treatment, PH continues to carry a mean 5‐year mortality of 33.3% to 46% depending on PH subtype,^[^ ^4^ ^]^ underscoring the necessity for a deeper understanding of its pathophysiology and the importance of effective management.

Right ventricular (RV) function is a critical determinant of survival in patients with pulmonary hypertension (PH). Cardiac magnetic resonance (CMR) has emerged as a gold‐standard imaging modality for the assessment of heart failure due to its ability to provide accurate and reproducible measures of ventricular function.^[^ ^5^ ^]^ The clinical importance of RV function assessment by CMR is powerfully underscored by a large meta‐analysis involving 1,938 PH patients. This analysis revealed that even a 1% absolute decrease in RV ejection fraction (RVEF) was associated with a 4.9% increase in the risk of clinical worsening within 22 months and a 2.1% rise in mortality risk over 54 months.^[^ ^6^ ^]^ However, the mechanisms underlying RV remodeling in response to PH remain incompletely understood. Recent studies suggest that cellular and molecular changes within the RV play a pivotal role in disease progression, including fibroblast (FBs) activation, cardiomyocyte hypertrophy, and metabolic changes.^[^ ^7^, ^8^, ^9^ ^]^ The activation of FBs induces cardiac fibrosis, reduces cardiac compliance, promotes cardiac remodeling, and ultimately leads to heart failure, rendering it a crucial factor in the development of heart failure.^[^ ^10^, ^11^, ^12^, ^13^ ^]^ In this context, cardiac FBs have emerged as crucial mediators of cardiac remodeling through their ability to modulate extracellular matrix (ECM) composition and cardiac fibrosis.

To elucidate the precise mechanism by which FB activation leads to RV remodeling, we employed single‐nucleus RNA sequencing (snRNA‐seq) to provide a comprehensive transcriptomic atlas of the RV in a murine model of PH induced by hypobaric hypoxia (HH). Our analysis revealed an upregulation of AXL protein level in RV FBs under PH conditions. As a member of the receptor tyrosine kinase family, AXL promotes tumor cell proliferation, migration, and invasion, as well as facilitates viral infection in pulmonary and bronchial epithelial cells.^[^ ^14^, ^15^ ^]^ Elevated AXL expression contributed to maladaptive RV remodeling by promoting cell proliferation, migration, and ECM production. These findings were further validated at the single‐nucleus level in both monocrotaline (MCT)‐induced PH and Sugen 5416/hypoxia (SuHx)‐induced PH in mice. Moreover, inhibition of AXL or its downstream signaling pathways ameliorated RV remodeling. Mechanistically, AXL was found to control FB activation via the PI3K‐AKT signaling pathway, which subsequently directed the nuclear localization of transcription factor nuclear factor I C (NFIC).

Together, this study provides comprehensive insights into the cellular and molecular mechanisms of RV remodeling in PH, underscores the significance of AXL in this pathological process, and lays the foundation for future investigations aimed at developing AXL‐targeted therapies against RV remodeling in PH.

## 2 Results

### 2.1 AXL Expression is Elevated in FBs of Remodeled RV Secondary to PH

**Figure 1:**
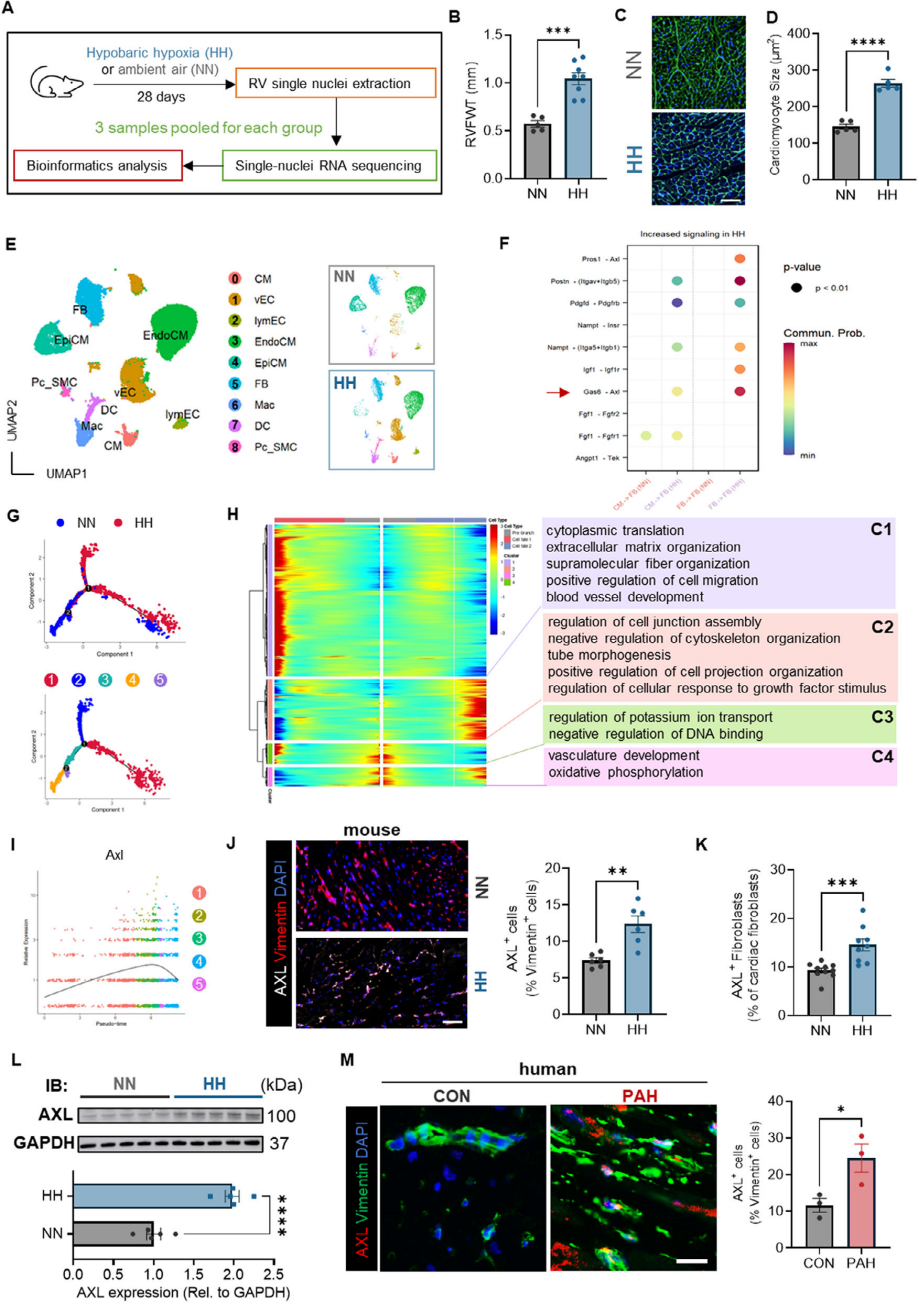
Elevated AXL expression levels in RV FBs of human and rodent PH. A) The experimental scheme involved harvesting RV tissues from PH mice under hypobaric hypoxia (HH) conditions and control mice in ambient air (NN) for the single‐nuclei RNA sequencing. B) RV free wall thickness (RVFWT) in PH mice (n = 5) and the control group (n = 8). C, D) Representative images of WGA staining (C) and the quantification of RV cardiomyocytes (D) in PH mice compared to the control group (n = 5/group); Scale bar: 50 µm. E) UMAP visualization of cell clustering across RV samples from both groups. F) Increased ligand‐receptor signaling in PH RV, with cardiomyocytes and FBs serving as signal‐sending (source) cells, and FBs acting as signal‐receiving (target) cells. G) Trajectory analysis of FBs showing gene signatures according to the group and cell state. H) Gene expression patterns in FBs of two transition states due to PH development were visualized in a heatmap and categorized into four clusters (C1‐C4) based on functional gene ontology. I) *Axl* expression intensity across trajectory states. J) Representative images of double immunofluorescent staining against AXL and Vimentin and quantification of AXL in FBs of RV tissues from HH or NN group (n = 6/ group); Scale bar: 50 µm. K) The proportion of AXL‐expressing FBs in RVs of the HH group (n = 10) or NN group (n = 9) by flow cytometry. L) AXL protein levels in the RVs of PH mice and the control group (n = 5/group). M) Representative images of double immunofluorescent staining against AXL and Vimentin and quantification of AXL in RV tissues from human PAH patients or controls; Scale bar: 20 µm. Data represent mean ± SEM. ^*^ *P* < 0.05, ^**^ *P* < 0.01, ^***^ *P* < 0.001, ^****^ *P* < 0.0001 compared to corresponding controls, as analyzed by unpaired *t* test.

As illustrated in **Figure** **1**A, mice were placed either in a HH chamber to establish a PH model (HH group) or in ambient air (NN group). We further investigated transcriptomic profiling alteration at the single‐nucleus level in RVs under PH conditions. Compared to control mice, PH mice exhibited elevated right ventricular systolic pressure (RVSP) (Figure S1A, Supporting Information) and RV remodeling, as indicated by increased right ventricular free wall thickness (RVFWT), higher cardiomyocyte score, and RV enlargement (Figure 1B–D; Figure S1B, Supporting Information). Meanwhile, RV fibrosis in PH mice was more pronounced than in the controls (Figure S2, Supporting Information).

Next, we performed snRNA‐seq on RVs from both groups. A total of 29 455 cells were annotated into 9 cell types according to the marker genes (Figure 1E; Figure S3, Supporting Information). PH RV exhibited greater cell communication intensity compared to control RV, with several pathways activated only in PH RV (Figure S4, Supporting Information). As cardiac FBs are the main contributors to cardiac fibrosis, we investigated the differences in cell communication related to the autocrine signaling of FBs and the paracrine signaling from cardiomyocytes to FBs between the PH RV and control RV. It turned out that *Axl*‐related pathways in FBs were significantly upregulated in the PH RV, regardless of whether it was in an autocrine manner or a paracrine manner with FBs as the target cells (Figure 1F). *Axl* expression in FBs of the PH RV was higher than in those of the control RV (Figure S5, Supporting Information). Pseudotime analysis of FBs from both groups showed a branched gene expression trajectory. FBs from the PH RV exhibited a distinct gene expression profile compared to those from the control RV, with state 2 FB subsets predominantly from the PH RV (Figure 1G; Figure S6, Supporting Information). The branched trajectory revealed the presence of two transition states due to PH development, along with the identification of differentially expressed genes (DEGs) distinguishing these states. Among the four distinct expression patterns identified, Cluster 1 exhibited a gene signature that was highly expressed in cell fate 1, demonstrating a heightened predisposition toward the HH group. This cluster was primarily associated with the regulation of the extracellular matrix and migration (Figure 1H). *Axl*, classified within Cluster 1 as depicted in Figure 1H, reached its highest expression at state 2 over pseudotime (Figure 1I).

Subsequent analysis confirmed an upregulation of AXL expression at the protein level in FBs from PH RV (Figure 1J,K). Furthermore, AXL protein level was elevated in PH RV homogenates relative to control RV samples (Figure 1L). Notably, there was a higher proportion of AXL‐positive cells in FBs from human PAH RV tissues compared to those from control tissues (Figure 1M). To cross‐validate *AXL* expression, we analyzed RV transcriptomic data from PH patients in the GEO database. *AXL* levels showed no significant difference between compensated and control RVs. In contrast, decompensated RVs exhibited marked *AXL* upregulation (GSE198618). This consistent trend was further supported by independent human RV tissue samples from dataset GSE240921 (Figure S7A, Supporting Information). Furthermore, transcriptional profiling was examined in two separate batches using the MCT‐induced PH rat model to examine compensated and decompensated RV states from dataset GSE240923. In the first batch, *Axl* expression remained unaltered in compensated RVs but was upregulated in the decompensated RVs. The second batch also revealed elevated *Axl* expression in both compensated and decompensated RVs, a pattern in line with the observations in the PAB model (Figure S7B,C, Supporting Information).

### 2.2 AXL in Cardiac FBs Confers RV Remodeling in PH

**Figure 2:**
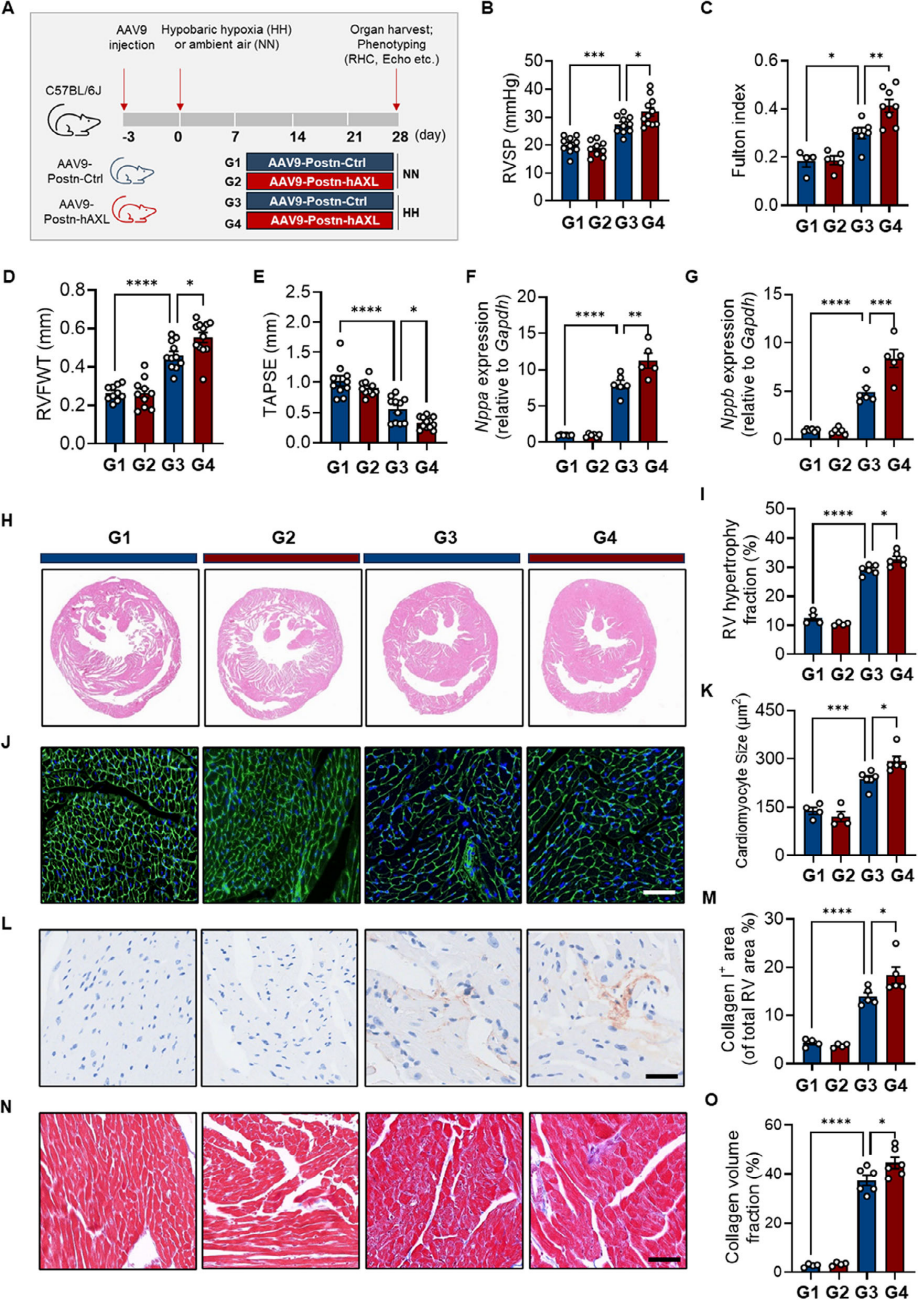
*Axl* overexpression in cardiac FBs aggravates RV remodeling in PH. A) Experimental scheme illustrating the treatment of mice with AAV9‐Postn‐hAXL (to overexpress AXL in cardiac FBs) or AAV9‐Postn‐Ctrl, followed by HH or NN conditions for four weeks (G1‐G4 groups as indicated). B–E) Right ventricular systolic pressure (RVSP; n = 9‐11/group) (B); Fulton index (n = 4‐8/group) (C); right ventricular free wall thickness (RVFWT; n = 10‐12/group) (D) and tricuspid annular plane systolic excursion (TAPSE; n = 10‐12/group) (E) in PH or control mice receiving AAV9‐Postn‐hAXL or AAV9‐Postn‐Ctrl. F, G) The transcriptional level of *Nppa* (F) and *Nppb* (G) in RV tissues from PH or control mice receiving AAV9‐Postn‐hAXL or AAV9‐Postn‐Ctrl (n = 5‐6/group). H–M) Representative HE staining (H) and assessment (I) of right ventricular size measured by right ventricular hypertrophy fraction; WGA staining (J) and cardiomyocyte size (K); IHC staining for collagen I (L) and the abundance of collagen deposition (M) measured by collagen I‐positive area; Masson staining for fibrosis N) and the quantification O) of fibrotic area in RV tissues from PH or control mice receiving AAV9‐Postn‐hAXL or AAV9‐Postn‐Ctrl, respectively. n = 4‐6/group; Scale bar: 50 µm. Data represent mean ± SEM. ^*^ *P* < 0.05, ^**^ *P* < 0.01, ^***^ *P* < 0.001, ^****^ *P* < 0.0001 compared to the indicated group, as analyzed by One‐way ANOVA test as appropriate.

To investigate the role of AXL overexpression in cardiac FBs during RV remodeling after HH exposure, we used AAV9‐Postn‐hAXL to overexpress AXL in heart FBs (**Figure** **2**A). AAV9‐Postn‐hAXL recipients showed elevated AXL in RV tissues and FBs (Figure S8, Supporting Information). Compared to AAV9‐Postn‐Ctrl recipients, AAV9‐Postn‐hAXL mice had higher RVSP (Figure 2B) and more severe RV remodeling after challenge, as evidenced by a higher Fulton index, increased RVFWT, and reduced TAPSE (Figure 2C–E). *Nppa* and *Nppb* were significantly higher at the transcriptional level in RVs from AAV9‐Postn‐hAXL mice compared to controls under HH conditions (Figure 2F,G). Furthermore, mice receiving AAV9‐Postn‐hAXL exhibited greater RV hypertrophy (Figure 2H,I), larger cardiomyocytes (Figure 2J,K), and increased deposition of Collagen I and fibrosis (Figure 2L–O) compared to AAV9‐Postn‐Ctrl mice after HH exposure. Following HH exposure, mice receiving AAV9‐Postn‐hAXL exhibited higher end‐systolic elastance (Ees), arterial elastance (Ea), and dp/dt_max_, along with lower dp/dt_min_, compared to AAV9‐Postn‐Ctrl mice (Figure S9, Supporting Information).

### 2.3 Knockdown of *Axl* in Cardiac FBs Alleviates PH‐Induced RV Remodeling

**Figure 3:**
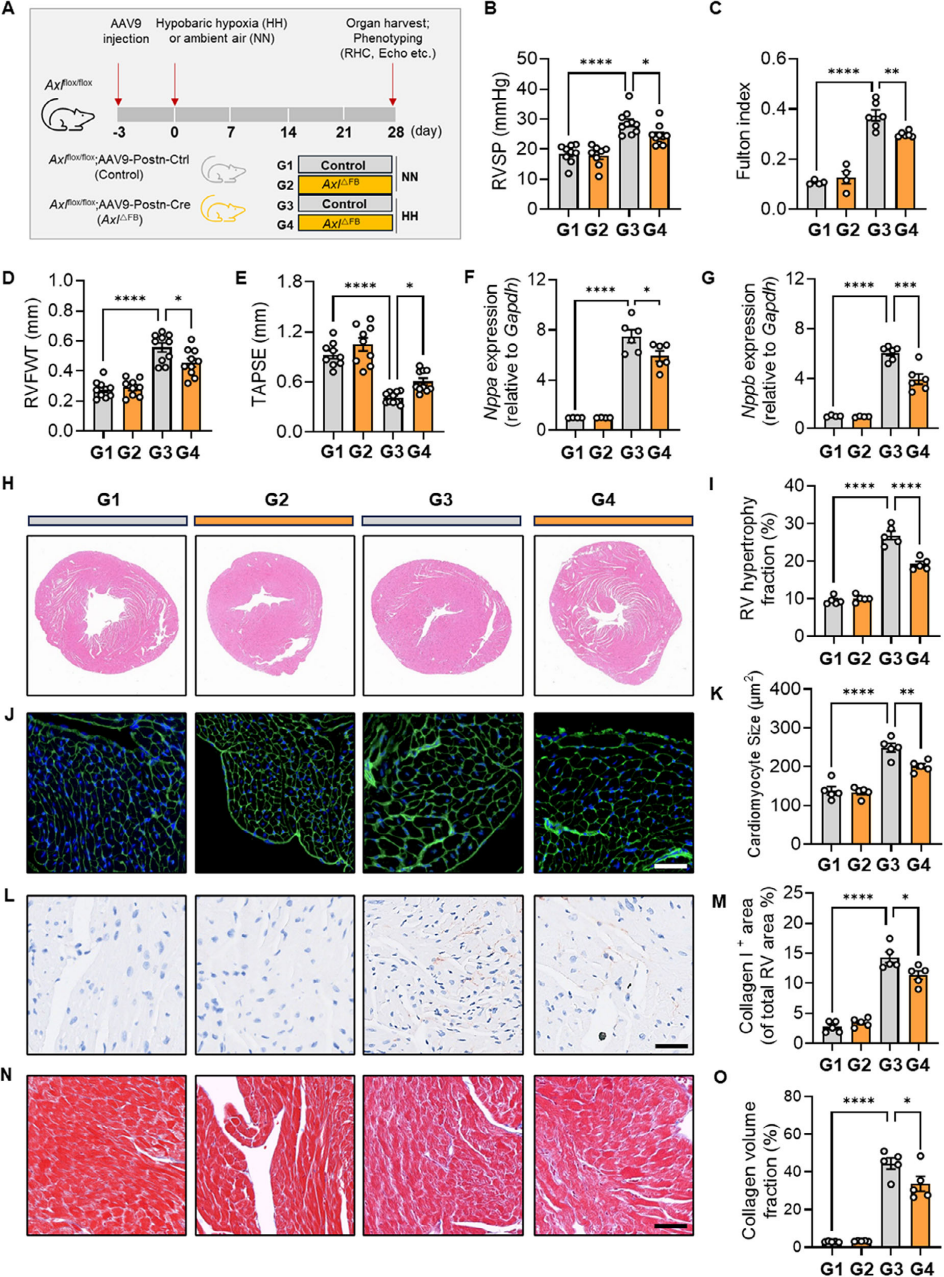
Knockdown of *Axl* in cardiac FBs ameliorates RV Remodeling in PH. A) Experimental scheme depicting the four‐week exposure of *Axl* ^flox/flox^ mice to HH or normoxic air (NN) after administration of either AAV9‐Postn‐Cre (to generate *Axl* ^ΔFB^ mice) or AAV9‐Postn‐Ctrl (control mice). The four experimental groups (G1‐G4) are indicated. B–E) RVSP (n = 8‐10/group) (B); Fulton index (n = 4‐6/group) (C); RVFWT (n = 9‐10/group) (D) and TAPSE (n = 9‐10/group) (E) in *Axl* ^ΔFB^ mice or controls under HH or NN conditions. F, G) The transcriptional level of *Nppa* (F) and *Nppb* (G) in RV tissues from G1 to G4 groups (n = 4‐6/group). H–M) Representative HE staining (H) and quantification (I) of right ventricular size measured by right ventricular hypertrophy fraction; WGA staining (J) and cardiomyocyte size by cardiomyocyte score (K); and IHC staining for collagen I (L) and the abundance of collagen deposition (M) measured by collagen I‐positive area; Masson staining for fibrosis N) and the quantification O) of fibrotic area in RV tissues from *Axl* ^ΔFB^ mice or controls under HH or NN conditions, respectively. n = 5/group; Scale bar: 50 µm. Data represent mean ± SEM. ^*^ *P* < 0.05, ^**^ *P* < 0.01, ^***^ *P* < 0.001, **** *P* < 0.0001 compared to the indicated group, as analyzed by One‐way ANOVA test.

To further investigate the role of FB *Axl* during PH‐induced RV remodeling, AAV9‐Postn‐Cre was introduced to *Axl* ^flox/flox^ mice to achieve cardiac FB‐specific deletion of *Axl*, thereby generating *Axl* ^△FB^ mice (**Figure** **3**A). This strategy dramatically reduced the *Axl* expression at the transcriptional level in RV tissues and at the protein level in RV FBs (Figure S10, Supporting Information). After exposure to HH challenge, *Axl* ^△FB^ mice had lower RVSP (Figure 3B), attenuated RV remodeling (lower Fulton index, decreased RVFWT, and elevated TAPSE; Figure 3C–E), and decreased levels of *Nppa* and *Nppb* in RVs compared to controls (Figure 3F,G). Furthermore, *Axl* ^△FB^ mice exhibited significantly reduced RV hypertrophy fraction, smaller cardiomyocytes, and less collagen I deposition and fibrosis (Figure 3H–O). These findings suggest that *Axl* knockout in cardiac FBs ameliorates PH‐induced RV remodeling.

### 2.4 AXL Augmentation Promotes the Proliferation, Migration, and ECM Production of FBs

**Figure 4:**
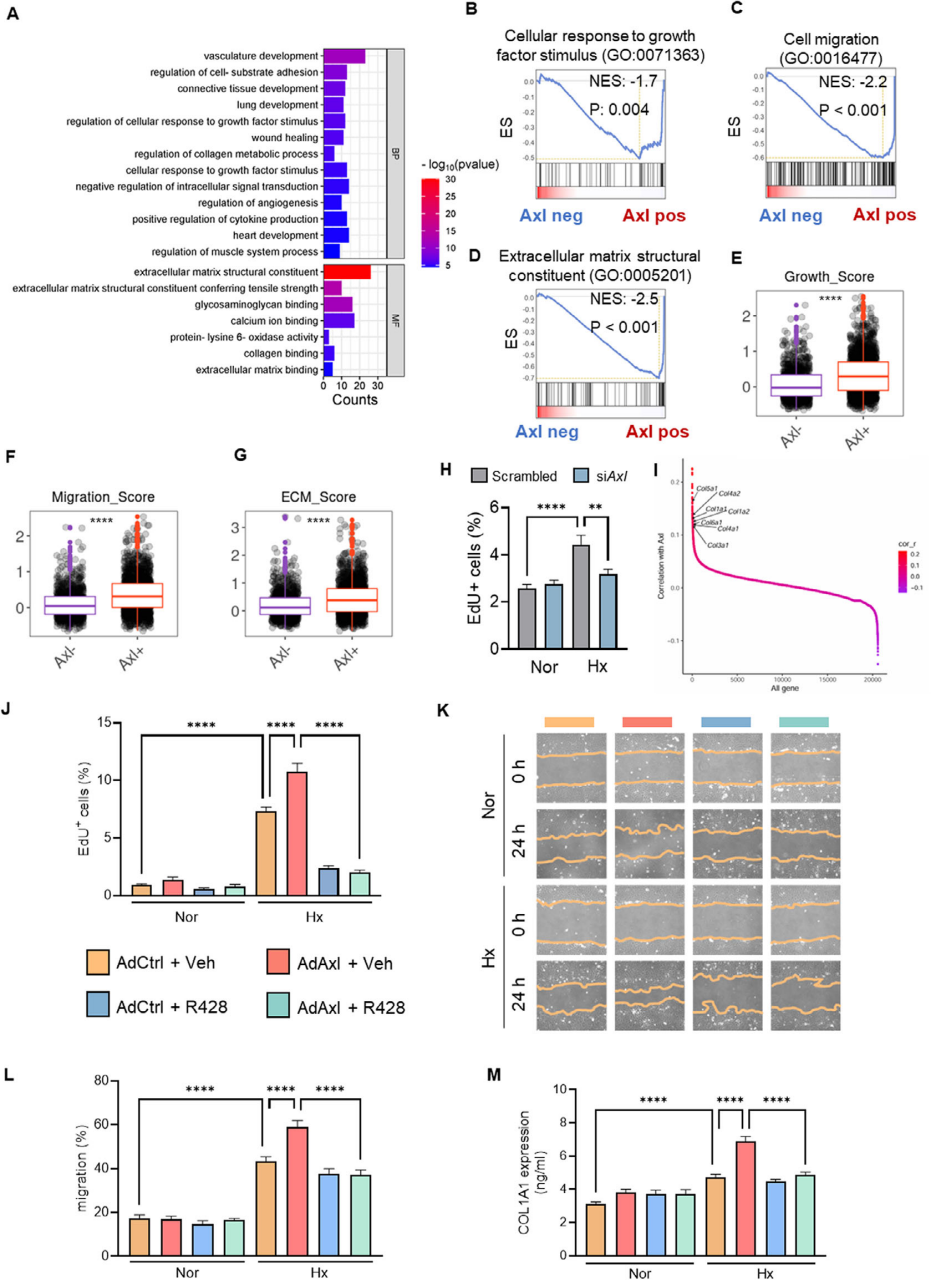
The impact of AXL expression on FB Function. A) The enrichment pathways of upregulated genes in *Axl*‐positive FBs compared to *Axl*‐negative FBs. BP, biological process; MF, molecular function. B–D) The GSEA analysis indicated enriched gene profiling related to cellular response to growth factor stimulus (B), cell migration (C), and ECM structural constituent (D) in *Axl*‐positive FBs. E–G) The differences in growth score (E), migration score (F), and ECM score (G) between *Axl*‐positive and *Axl*‐negative FBs. H) The effect of *Axl* silencing on FB proliferation in response to hypoxia for 24 h, measured by EdU assay (distributed in four independent experiments). I) Correlation analysis reveals a significant positive relationship between *Axl* expression and collagen‐related genes in FBs. J–M) The effect of AXL inhibitor R428 on proliferation (J), migration (K, L), and synthesis of collagen COL1A1 (M) of FBs infected with AdAxl or AdCtrl in the presence or absence of R428 under hypoxic conditions (distributed in three independent experiments). Data represent mean ± SEM. ^**^ *P* < 0.01, ^***^ *P* < 0.001 compared to indicated group, as analyzed by One‐way ANOVA test.

To explore the impact of AXL on FB function, we categorized FBs into *Axl*‐expressing and *Axl*‐deficient FBs. Axl‐expressing FBs showed upregulated DEGs enriched in wound healing, growth factor response, and ECM processes (**Figure** **4**A). GSEA analysis revealed pathways linked to proliferation, migration, and ECM constitution were more active in Axl‐expressing FBs (Figure 4B–D). FB activation scores (growth, migration, ECM) were higher in PH RV than in controls (Figure S11A–C, Supporting Information). Next, we isolated primary mouse cardiac FBs of high purity for further functional assays (Figure S12, Supporting Information). Following hypoxia exposure, these isolated FBs exhibited a marked upregulation of collagen‐related proteins (Figure S13, Supporting Information). Intriguingly, the scores related to proliferation, migration, and ECM components were also higher in *Axl*‐expressing FBs than in *Axl*‐deficient FBs (Figure 4E–G), highlighting AXL's role in FB activation. Furthermore, we found that *Axl* deficiency attenuated FB proliferation post‐hypoxia (Figure 4H). The knockdown efficiency of *Axl* was validated (Figure S14, Supporting Information). We next examined the genes positively correlated with *Axl*, most of which were collagen‐related genes (Figure 4I) and enriched in pathways including collagen biosynthesis and modifying enzymes (Figure S15, Supporting Information).

R428 can bind to AXL, preventing its phosphorylation and subsequently inhibiting the downstream signaling pathway.^[^ ^16^ ^]^ *Axl* overexpression (Figure S16A, Supporting Information) promoted FB proliferation and migration in response to hypoxia, which could be restored by R428 treatment (Figure 4J–L). Moreover, the increase in collagen synthesis induced by *Axl* upregulation was also effectively blunted by R428 (Figure 4M; Figure S16B,C, Supporting Information). These results indicate that AXL is critical for FB activation, thus contributing to RV remodeling.

### 2.5 Expression and Functional Role of AXL in RVs of MCT‐Induced PH or SuHx‐Induced PH

To further scrutinize the alteration of *Axl* in remodeled RVs due to PH, we also employed snRNA‐seq on the RV of MCT‐administered PH rats and that of SuHx‐induced PH mice. MCT‐induced rats exhibited higher RVSP, increased RV fibrosis, and larger cardiomyocytes (Figure S17A–C, Supporting Information). A total of 31 245 cells were categorized into nine cell types, and the distribution of which was comparable between the MCT‐treated and saline‐treated groups (Figure S17D, Supporting Information). *Axl* and *Col1a1* were significantly elevated, primarily in FBs of the MCT group (Figure S17E, Supporting Information). In line with the findings in human RVs, a higher proportion of AXL‐positive cells was observed in FBs from MCT RVs (Figure S17F, Supporting Information). Additionally, FB activation scores (growth, migration, ECM) were higher in MCT RVs (Figure S17G, Supporting Information) and further elevated in *Axl*‐expressing FBs compared to their *Axl*‐deficient counterparts (Figure S17H, Supporting Information). Of note, AXL expression in cardiac FBs was significantly upregulated in the RVs at the compensated state (3 weeks post‐MCT administration) and exhibited a further increase in the RVs at the decompensated state (5 weeks post‐MCT administration) (Figure S18, Supporting Information).

To further confirm the role of AXL overexpression in cardiac FBs during RV remodeling after MCT challenge, we also used AAV9‐Postn‐hAXL to overexpress AXL in cardiac FBs (Figure S19A, Supporting Information). Compared to AAV9‐Postn‐Ctrl recipients, AAV9‐Postn‐hAXL rats had higher RVSP, RVFWT, and transcriptional levels of *Nppa* and *Nppb* in the RVs, along with reduced TAPSE after MCT administration (Figures S19B–F, Supporting Information). Furthermore, rats receiving AAV9‐Postn‐hAXL exhibited greater RV hypertrophy (Figure S19G,H, Supporting Information), larger cardiomyocytes (Figure S19I,J, Supporting Information), and increased deposition of Collagen I and fibrosis (Figure S19K–N, Supporting Information) compared to AAV9‐Postn‐Ctrl rats after MCT challenge.

Similarly, higher RVSP, increased RV fibrosis, and larger cardiomyocytes were documented in SuHx mice (Figure S20A–C, Supporting Information). Among 29 844 cells clustered into eight cell types, *Axl* and *Col1a1* expressions were also primarily upregulated in FBs of the SuHx group (Figure S20D,E, Supporting Information). A higher proportion of AXL‐positive cells was observed in FBs from SuHx RVs (Figure S20F, Supporting Information), and FB activation scores were higher in SuHx RVs and elevated in *Axl*‐expressing FBs compared to *Axl*‐deficient FBs (Figure S20G,H, Supporting Information). Temporal profiling of AXL expression was further investigated. The results revealed a significant upregulation of AXL after two weeks of suHx exposure, which was further enhanced after four weeks (Figure S21, Supporting Information), indicating a time‐dependent increase in AXL expression.

To further elucidate the role of AXL in cardiac FBs during RV remodeling in response to suHx challenge, *Axl^flox/flox^*; PostnCre (*Axl* ^FB‐KO^) mice were generated (Figure S22A, Supporting Information). Following suHx exposure, *Axl* ^FB‐KO^ mice had attenuated RV remodeling, as evident by reduced RVSP, decreased RVFWT, lower transcriptional levels of *Nppa* and *Nppb* in the RVs, and improved TAPSE, compared to *Axl* ^flox/flox^ mice (Figure S22B–F, Supporting Information). *Axl* ^FB‐KO^ mice also showed diminished RV hypertrophy (Figure S22G,H, Supporting Information), smaller cardiomyocytes (Figure S22I,J, Supporting Information), along with reduced Collagen I deposition and fibrosis (Figure S22K–N, Supporting Information) relative to control mice.

In addition, AXL expression was significantly elevated in the lungs of PH mice induced by HH or suHx challenge (Figure S23A,B, Supporting Information) as well as in PH rats after MCT administration, compared to their corresponding control groups (Figure S23C, Supporting Information).

### 2.6 The Role of AXL in Cardiac FBs in PAB‐Induced RV Remodeling

To further investigate the role of AXL overexpression in cardiac FBs during RV remodeling following PAB, we employed AAV9‐Postn‐hAXL to achieve cardiac FB‐specific overexpression of AXL (Figure S24A, Supporting Information). Compared to AAV9‐Postn‐Ctrl recipients, mice receiving AAV9‐Postn‐hAXL exhibited no difference in RVSP, velocity times integral (VTI), and cardiac output (CO), but increased RVFWT, and higher transcriptional levels of *Nppa* and *Nppb* in RV tissues, accompanied by reduced TAPSE after PAB (Figure S24B–G, Supporting Information). Furthermore, AAV9‐Postn‐hAXL‐treated mice developed more pronounced RV hypertrophy (Figure S24H,I, Supporting Information), larger cardiomyocyte size (Figure S24J,K, Supporting Information), and increased deposition of Collagen I and fibrosis (Figure S24L–O, Supporting Information) compared to control mice following PAB procedure.

To evaluate the therapeutic potential of AXL inhibition, we administered R428 to mice one week after PAB surgery (Figure S25A, Supporting Information). R428 treatment did not alter RVSP, VTI, or CO (Figure S25B,E,F, Supporting Information), but led to improved RV function and structure. Specifically, R428‐treated mice exhibited reduced RVFWT, improved TAPSE, and lower transcriptional levels of *Nppa* and *Nppb* compared to vehicle‐treated controls following PAB (Figure S25C,D,G, Supporting Information). Additionally, R428 administration attenuated RV hypertrophy, cardiomyocyte size, collagen I deposition, and fibrosis relative to vehicle‐treated mice with PAB surgery (Figure S25H–O, Supporting Information).

### 2.7 Suppression of *Nfic* Mitigates FB Activation Triggered by *Axl* Upregulation

**Figure 5:**
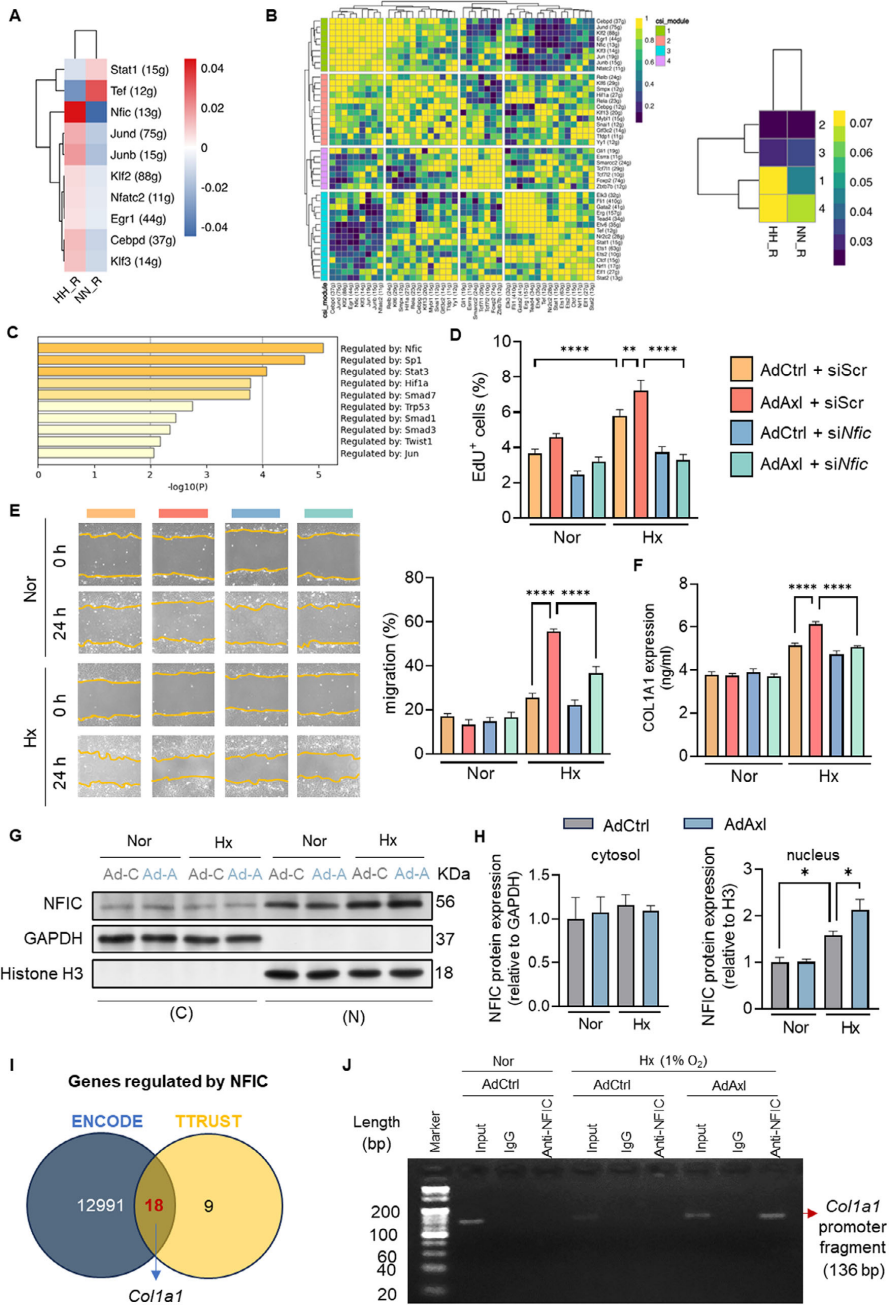
Suppression of NFIC rescues FB activation in response to AXL upregulation. A) The differentially expressed transcription factors (TFs) in FBs from PH RV relative to control RVs. The letter “g” is used as the abbreviation for genes regulated by transcription factors, and the number represents the number of genes. B) The combinatorial patterns of expressed TFs based on the connection specificity index (CSI) using SCENIC. C) Predicted TFs regulating the genes in a positive relationship with the *Axl* expression levels in FBs. D) The effect of *Nfic* knockdown on the proliferation of FBs infected with AdAxl or AdCtrl under hypoxic conditions, measured by EdU incorporation assay (distributed in six independent experiments). E) Representative images and quantification of scratch assays showed decreased migratory ability in FBs with *Axl* overexpression upon *Nfic* suppression (distributed in three independent experiments). F) The effect of *Nfic* knockdown on the synthesis of collagen COL1A1 in FBs infected with AdAxl or AdCtrl under hypoxic conditions, as measured by ELISA (distributed in three independent experiments). G, H) Representative blotting image (G) and quantification (H) of NFIC protein levels in cytosolic and nuclear fractions from FBs infected with either AdAxl or AdCtrl under hypoxic or normoxic conditions (distributed in four independent experiments). Protein levels were normalized to GAPDH in the cytosol and histone H3 in the nucleus. I) Venn diagram illustrating 18 genes commonly regulated by *Nfic*, identified via ENCODE and TRUST databases, with *Col1a1* featured as a key target. J) The identification of NFIC's binding to the *Col1a1* promoter in FBs with *Axl* overexpression in response to hypoxia by CHIP‐PCR (distributed in three independent experiments). The bands from the Input sample serve as the positive control for the ChIP‐PCR. Data represent mean ± SEM. ^*^ *P* < 0.05, ^**^ *P* < 0.01, ^***^ *P* < 0.001, ^****^ *P* < 0.0001 compared to the indicated group, as analyzed by One‐way ANOVA test.

To determine the regulatory program driving FB activation, we analyzed transcription factor (TF) expression at the single‐cell level in FBs from PH models compared to controls. *Nfic* emerged as the most differentially expressed TF, showing significantly higher levels in RV FBs from the HH group (**Figure** **5**A). To systematically delineate the combinatorial patterns of expressed TFs, we assessed the similarity of binarized regulon activity scores for each pair of regulons using the connection specificity index (CSI). This assessment grouped 43 regulons into four primary TF modules, with CSI module 1 being the most effective at distinguishing FBs from PH RV and control RV FBs. Notably, *Nfic* was among the regulons in the CSI module 1 (Figure 5B). Further analysis revealed *Nfic* as the top TF regulating genes positively correlated with *Axl* expression (Figure 5C). Silencing *Nfic* effectively blunted FB proliferation under hypoxia (Figure S26A,B, Supporting Information) and attenuated the enhanced FB activation induced by *Axl* overexpression (Figure 5D–F; Figure S27A,B, Supporting Information).

Previous studies have confirmed that NFIC primarily functions through nuclear translocation.^[^ ^17^, ^18^, ^19^ ^]^ Here, we found a significant increase in the nuclear protein levels of NFIC in AdAxl‐infected FBs under hypoxia compared to those in the AdCtrl group (Figure 5G,H). To identify downstream targets of NFIC, we screened gene lists from the ENCODE and TRUST databases, identifying 18 common genes (Figure 5I), including *Col1a1*, which positively correlated with *Axl* expression. Hypoxia increased NFIC binding to the *Col1a1* promoter in *Axl*‐overexpressing FBs (Figure 5J). These findings suggest that NFIC acts as a downstream TF of AXL.

### 2.8 Inhibition of the PI3K‐AKT Pathway Alleviates FB Activation in Response to AXL

**Figure 6:**
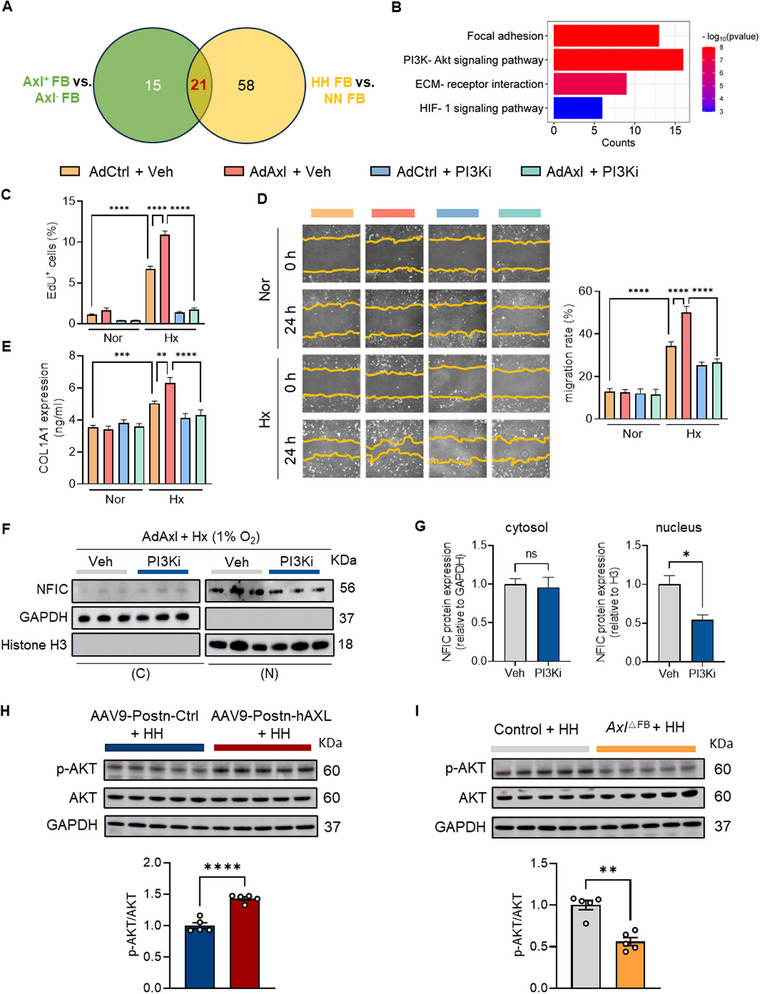
Inhibition of the PI3K‐AKT pathway alleviates FB activation in response to AXL. A) The analysis of enriched pathways for DEGs revealed 21 common pathways between *Axl*‐positive and *Axl*‐negative FBs, as well as between PH and control FBs. B) Major differential pathways (as in A) were visualized in a bar plot. C) EdU incorporation assay showed cell proliferation increased under hypoxia in *Axl*‐overexpressing FBs, and this effect was reversed by a PI3K‐Akt inhibitor (LY294002) (distributed in three independent experiments). D) The scratch assay demonstrated *Axl* overexpression in FBs under hypoxia enhanced cell migration, an effect that could be counteracted by PI3K inhibition (distributed in three independent experiments). E) COL1A1 expression at the protein level was elevated in Axl‐overexpressing FBs, and this increase was blunted by PI3K inhibition (distributed in three independent experiments). F, G) Representative blotting image (F) and quantification (G) of NFIC protein levels in cytosolic and nuclear fractions from FBs infected with either AdAxl or AdCtrl under hypoxic conditions in the presence or absence of PI3K inhibitor (distributed in three independent experiments). Protein levels were normalized to GAPDH in the cytosol and histone H3 in the nucleus. H) AKT phosphorylation in RVs from either AAV9‐Postn‐hAXL or AAV9‐Postn‐Ctrl recipients under HH conditions (n = 5/group). I) AKT phosphorylation in RVs from either *Axl* ^ΔFB^ mice or control mice under HH conditions (n = 5/group). Data represent mean ± SEM. ^*^ *P* < 0.05, ^**^ *P* < 0.01, ^***^ *P* < 0.001, ^****^ *P* < 0.0001 compared to indicated group, as analyzed by One‐way ANOVA test; ns indicates not significant.

To further investigate the mechanism by which AXL upregulation drives NFIC nuclear translocation, we screened the DEGs between *Axl*‐expressing and *Axl*‐deficient FBs followed by the enrichment analysis. Subsequently, we identified 21 common pathways enriched in PH RV FBs compared to controls (**Figure** **6**A), with focal adhesion, PI3K‐Akt signaling, ECM‐receptor interaction, and HIF‐1 signaling pathway being the most prominent (Figure 6B). Treatment of *Axl* overexpressing FBs with PI3K or HIF‐1 inhibitors reversed the enhanced proliferation, migration, and collagen synthesis induced by *Axl* overexpression (Figure 6C–E; Figures S28A,B, and S29A–E, Supporting Information), whereas the HIF2α inhibitor had no effect (Figure S30A–E, Supporting Information). Of note, PI3K inhibition reduced NFIC nuclear protein levels under hypoxia (Figure 6F,G). Additionally, enhanced AKT phosphorylation was displayed in RVs from AAV9‐postn‐hAXL recipients under HH conditions (Figure 6H), while *Axl* knockout in cardiac FBs decreased AKT phosphorylation (Figure 6I). Overall, our data suggest that AXL triggers FB activation in a PI3K signaling‐dependent manner.

### 2.9 AXL‐Exacerbated RV Remodeling in PH is Rescued by R428 or LY294002

**Figure 7:**
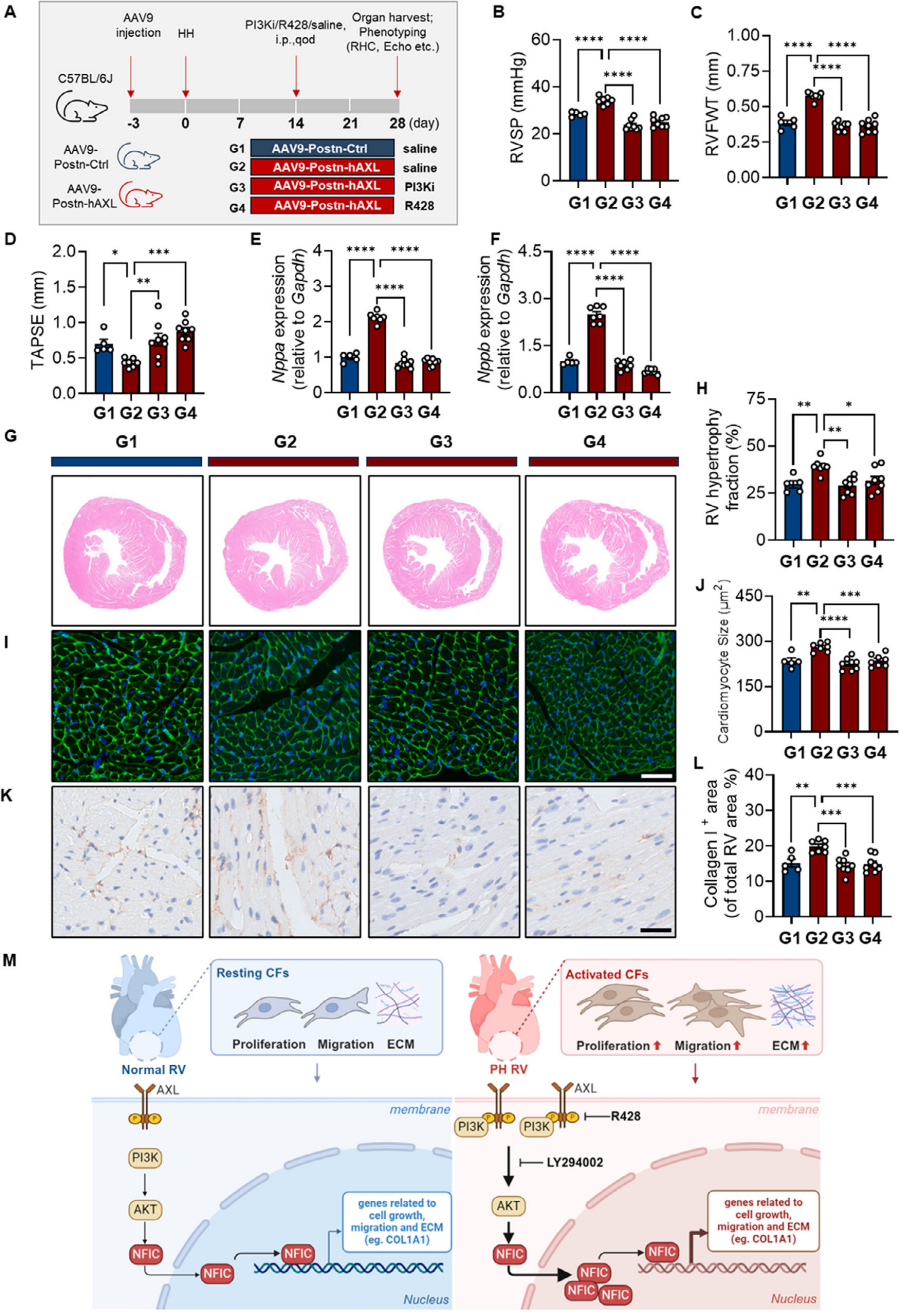
AXL‐exacerbated RV remodeling in PH is rescued by R428 or LY294002. A) Experimental scheme outlining the treatment protocol for mice that received either AAV9‐Postn‐hAXL or AAV9‐Postn‐Ctrl, followed by administration of LY294002 (a PI3K inhibitor) or R428 (an AXL inhibitor) every other day for two weeks, starting two weeks after HH exposure (G1‐G4 groups as indicated). B–D) RVSP (n = 5‐9/group) (B); RVFWT (n = 5‐9/group) (C) and TAPSE (n = 5‐9/group) (D) in mice receiving either AAV9‐Postn‐hAXL or AAV9‐Postn‐Ctrl, followed by administration of LY294002 or R428 under HH conditions. E, F) The transcriptional level of *Nppa* (E) and *Nppb* (F) in RV tissues from G1 to G4 groups (n = 5‐9/group). G, H) Representative HE staining images (G) and quantification analysis (H) revealed PI3K blockade or R428 mitigated RV hypertrophy fraction in AAV9‐Postn‐hAXL recipients exposed to HH conditions (n = 5–8/group). I, J) Representative WGA staining images (I) and quantification (J) demonstrated a reduced cardiomyocyte size in AAV9‐Postn‐hAXL recipients exposed to HH conditions (n = 5–8/group). K, L) Representative IHC staining images (K) and quantification (L) showed a reduction in Collagen I‐positive areas in RV tissue of mice receiving AAV9‐Postn‐hAXL under HH conditions (n = 5–8/group). M) Synopsis of proposed mechanism: In the context of PH, excessive AXL signaling activates the PI3K‐AKT pathway, facilitating the nuclear translocation of NFIC. This process promotes the transcription of targeted genes that enhance FB proliferation, migration, and collagen synthesis, contributing to RV remodeling. This remodeling can be mitigated by blocking the PI3K pathway with LY294002 and using R428 (Created in BioRender. Yan, Y. (2025) https://BioRender.com/s34e956). Data represent mean ± SEM. ^*^ *P* < 0.05, ^**^ *P* < 0.01, ^***^ *P *< 0.001, ^****^ *P* < 0.0001 compared to indicated group, as analyzed by One‐way ANOVA test; Scale bar: 50 µm.

To address whether PI3K or AXL inhibition could attenuate RV remodeling in vivo, mice receiving AAV9‐Postn‐hAXL or AAV9‐Postn‐Ctrl were treated with LY294002 (PI3K inhibitor) or R428 (AXL inhibitor) starting two weeks post HH exposure (**Figure** **7**A). AAV9‐Postn‐hAXL mice exhibited worsened hemodynamics, which were improved by LY294002 or R428 (Figure 7B). Notably, both inhibitors led to reduced RVFWT, improved TAPSE, and lower *Nppa* and *Nppb* expression at mRNA levels in AAV9‐Postn‐hAXL mice (Figure 7C–F). Additionally, both inhibitors reduced RV hypertrophy, cardiomyocyte size, and collagen I deposition (Figure 7G–L). Collectively, our findings demonstrate that AXL on FBs triggers NFIC translocation in a PI3K‐AKT signaling‐dependent fashion, which engages their proliferation, migration, and collagen synthesis to fuel RV remodeling in PH (Figure 7M).

## 3 Discussion

Our endeavor to disambiguate the mechanisms underlying RV remodeling in the context of PH uncovered that *Axl* was elevated in cardiac FBs within the RVs at single‐nucleus resolution. In silico analyses further revealed that *Axl*‐expressing FBs from PH RVs exhibited higher proliferation, migration, and ECM capabilities. Functional studies confirmed that *Axl* overexpression in cardiac FBs exacerbated RV remodeling in both PH models and PAB modes, whereas *Axl* knockdown in cardiac FBs alleviated PH‐induced RV remodeling. Mechanistically, we found that AXL signals through the PI3K‐AKT pathway, licensing the nuclear translocation of NFIC, which in turn promotes the transcription of its targets such as *Col1a1*, supporting FB proliferation, migration, and collagen synthesis – key drivers of RV remodeling in PH. Accordingly, inhibition of PI3K or treatment with R428 mitigated AXL‐exacerbated RV remodeling in PH. R428 treatment also ameliorated RV remodeling in the PAB mouse model. This study provides a comprehensive understanding of how AXL contributes to the pathological changes in remodeled RVs in the scenario of PH, providing a foundation for potential therapeutic interventions.

Our study highlights that the activation of FBs upon AXL activation provides critical insights into the cellular processes driving RV remodeling. The receptor tyrosine kinase AXL is a transmembrane protein that belongs to the tyrosine kinase family.^[^ ^14^ ^]^ It primarily participates in various biological processes, including cell proliferation, migration, and immune regulation. Under physiological conditions, AXL is acknowledged as an essential element for the maintenance of cellular activity in environments such as tissue repair and embryonic development.^[^ ^20^, ^21^ ^]^ However, aberrant activation of AXL can significantly enhance cell migration and invasion capabilities, which are closely associated with cancer metastasis.^[^ ^22^ ^]^ Consequently, AXL is often overexpressed in several tumors and linked to enhanced invasiveness and poorer prognosis.^[^ ^23^, ^24^ ^]^ Previous studies also revealed the overexpression of AXL in FB‐like cells in osteoarthritis and rheumatoid arthritis,^[^ ^25^, ^26^ ^]^ however, the specific role of AXL in cardiac FBs remains unclear. At the molecular level, AXL primarily promotes tumor cell proliferation and migration by activating downstream pathways such as RAS‐RAF‐MEK‐ERK and PI3K‐AKT‐mTOR,^[^ ^27^ ^]^ at least in part consistent with our study. Notably, our findings position AXL as a central regulator of RV remodeling, not only in response to PH but also in the context of pressure overload induced by PAB. The observation that AXL inhibition ameliorated RV dysfunction and remodeling in the PAB model is particularly significant. Since the PAB model induces RV failure independently of pulmonary vascular pathology, this demonstrates conclusively that AXL drives adverse cardiac remodeling through mechanisms intrinsic to the heart. Overall, all these findings and consistent upregulation of AXL—observed in both human and experimental PH RV tissues— suggest that targeting AXL may represent a promising therapeutic strategy for mitigating RV remodeling in PH.

The PI3K/AKT signaling pathway, as one of the core pathways regulating cell growth, proliferation, metabolism, and survival,^[^ ^28^ ^]^ can directly regulate its downstream targets, such as mTOR and HIF‐1α, and is involved in the development of various diseases. Currently, research on the PI3K/AKT pathway is receiving considerable attention in the fields of tumors and fibrotic diseases. Studies have shown that the activation of the PI3K/AKT pathway not only promotes the excessive proliferation of bladder cancer cells but also induces the transformation of FBs into tumor‐associated FBs, which in turn leads to the upregulation of interleukin‐6 secretion, supporting the formation of the tumor immune microenvironment and promoting tumor growth.^[^ ^29^ ^]^ In idiopathic pulmonary fibrosis, the PI3K/AKT pathway contributes to FB activation within lung tissues, thereby exacerbating fibrotic progression. In contrast, STX11 inhibits the PI3K/AKT pathway by enhancing autophagy, leading to subsequent suppression of FB activation and modulation of fibrosis.^[^ ^30^ ^]^ The previous studies support the notion that the PI3K/AKT pathway may regulate the activation phenotype of cardiac FBs. Our findings suggest that targeted inhibition of PI3K‐AKT signaling may represent a promising therapeutic strategy for mitigating RV remodeling in PH.

NFIC is acknowledged for its roles in regulating gene transcription, tissue development and differentiation, and DNA repair.^[^ ^31^, ^32^, ^33^ ^]^ NFIC can promote the proliferation of lung cancer cells in progressive‐stage lung cancer by binding to the transcriptional regions of proliferation‐related genes. Our study highlights the crucial role of transcriptional regulation in RV remodeling and identifies NFIC as a downstream target of AXL, which dictates NFIC's translocation and subsequent binding to the promoter region of *Col1a1*, thereby enhancing ECM synthesis, a mechanism that has not been previously documented. Our discoveries underscore that targeting NFIC is of paramount importance for developing future therapies to address RV remodeling in PH.

Several limitations should be acknowledged. First, our findings are primarily derived from in vitro and rodent models, which may not fully replicate human physiology. Second, the sample size for snRNA‐seq was relatively small, potentially limiting the statistical power and generalizability of our findings. Nonetheless, the observed upregulation of AXL in cardiac FBs from RVs of human PAH patients and further functional studies, both in vivo and in vitro, support the significance of AXL in RV remodeling.

In summary, our study elucidates that AXL signals through the PI3K‐AKT pathway, facilitating the nuclear translocation of NFIC. This process may promote the transcription of genes related to FB activation, ultimately contributing to RV programming. Our findings provide a mechanistic basis for targeting AXL and PI3K‐AKT signaling to combat RV remodeling in PH.

## 4 Experimental Section

### Chemicals and Reagents

Monocrotaline (MCT) was purchased from Sigma–Aldrich (Cat# C2401). Sugen 5416 (SU5416) was also purchased from Sigma–Aldrich (Cat# S8442). AXL inhibitor R428 (Cat# HY‐15150) and PI3K inhibitor LY294002 (Cat# HY‐10108) were purchased from MedChemExpress LLC. HIF‐1α inhibitor KC7F2 (Cat# 4324) and HIF‐2α inhibitor TC‐S 7009 (Cat# 5243) were purchased from Tocris Bioscience.

### Human Samples

The use of human RV tissues was approved by the Ethics Committee of the Shanghai Children's Medical Center (SCMCIRB‐K2024081‐1). In brief, right heart tissue from three patients with pulmonary arterial hypertension (PAH, Group I PH) and three non‐PAH donors was harvested (Table S1, Supporting Information). RV tissues were fixed and paraffin‐embedded. The sections were later used for immunofluorescence staining in this study. The patients or family guardians of the donors all signed informed written consent.

### Animals—Hypobaric Hypoxia (HH) Induced PH in Mice

To investigate the effect of *Axl* deficiency in FB on RV remodeling, *Axl* floxed (*Axl* ^f/f^) mice were purchased from Cyagen Company. Eight‐week‐old male *Axl* ^f/f^ mice received the injection of AAV9‐Postn‐Cre‐ZsGreen (0.9×10^11^ VG) via the medial canthus vein. *Axl* ^f/f^ mice received the same dosage injection of AAV9‐Postn‐ZsGreen as controls.

To investigate the effect of AXL augmentation in FB on RV remodeling, 50 µL of AAV9‐Postn‐hAXL at a dosage of 0.7×10^11^ VG was delivered into the medial canthus vein of male C57BL/6J mice (Beijing Vital River Laboratory Animal Technology Co., Ltd.). Control male mice were injected with 50 µL of AAV9‐Postn‐Ctrl.

For hypobaric hypoxia induction, three days post AAV9 injection, mice were placed in a hypobaric hypoxia chamber (Shanghai TOW Intelligent Technology Co., Ltd, ProOX‐850; 10% O_2_) for 4 weeks. For normoxic experiments, mice were maintained at ambient temperature for 4 weeks post‐AAV9 injection.

To investigate the potential of R428 or LY294002 (PI3K inhibitor) treatment, male C57BL/6J mice were treated with AAV9‐Postn‐hAXL or AAV9‐Postn‐Ctrl as described above. Three days post AAV9 delivery, mice were then placed in a hypobaric hypoxia chamber or maintained at ambient for 2 weeks, followed by intraperitoneal injection of R428 (25 mg kg^−1^) or LY294002 (50 mg kg^−1^), or saline every other day for another 2 weeks.

At the end of the experiments, echocardiography, right heart catheterization, and tissue harvest were performed.

### Animals—Sugen 5416 and Hypoxia (SuHx) Induced PH in Mice

Eight‐week‐old male C57BL/6J mice (18–20 g) were randomized into two groups. The SU5416‐treated mice were placed in a hypoxic chamber with 10% oxygen for 3 weeks, during which they received weekly subcutaneous injections of SU5416 (20 mg kg^−1^), followed by re‐oxygenation for 1 week. Control mice received the same amount of subcutaneous injection of saline and were maintained at ambient temperature for 4 weeks. Phenotyping was assessed by right heart catheterization and echocardiography. Tissue collection was performed at week 2 and week 4 post‐first dose of SU5416 or saline injection. To investigate the effect of FB‐specific genetic ablation of *Axl* on RV remodeling, we generated *Axl* ^flox/flox^;Postn‐Cre mice by crossing *Axl* ^flox/flox^ mice with Postn‐Cre mice. *Axl* ^flox/flox^ littermates without Cre were used as controls. Following a 5‐day course of tamoxifen induction (75 mg/kg/day, i.p.), mice were subjected to chronic hypoxia for 3 weeks. During this period, they received weekly subcutaneous injections of SU5416 (20 mg/kg). Normoxic control mice were maintained at ambient conditions. Tissue collection was performed at end of experiment. RV tissues were frozen at −80 °C before further use.

### Animals—Pulmonary Artery Banding (PAB) Induced RV Remodeling in Mice

Eight‐week‐old male C57BL/6J mice were anesthetized and intubated for mechanical ventilation. A 5‐mm transverse skin incision was made 2 mm from the left sternal border and 2 mm caudal to the axilla. A thoracotomy was performed at the second intercostal space. The pulmonary trunk was carefully dissected from surrounding tissue, and a tunnel was created beneath it using an L‐shaped vessel probe. A 27G introducer needle was placed to occlude ≈60–70% of the luminal diameter. Sham‐operated mice underwent the same procedure without clip placement. The thoracic cavity was closed in layers, and the lungs were re‐inflated prior to recovery.

### Animals—Monocrotaline (MCT)‐Induced PH in Rats

Eight‐week‐old male Sprague‐Dawley rats (180–200 g) were purchased from Beijing Vital River Laboratory Animal Technology Co., Ltd. and randomized into two groups. Rats were either treated with a one‐dose intraperitoneal injection of MCT (60 mg kg^−1^) or saline. Phenotyping was assessed 4 weeks later. To investigate the effect of AXL augmentation in FB on RV remodeling, 200 µL of AAV9‐Postn‐hAXL at a dosage of 0.7×10^11^ VG was delivered into the medial canthus vein of male Sprague–Dawley rats. Control male rats were injected with 200 µL of AAV9‐Postn‐Ctrl. Echocardiography and right heart catheterization were performed 4 weeks later. Tissues were harvested and frozen at −80 °C before further use. To recapitulate the compensated and decompensated phases of RV adaptation, rats were administered a single dose of MCT and evaluated at 3 weeks (compensated state) and 5 weeks (decompensated state) post‐injection.

All animal experiments were conducted according to Shanghai Children's Medical Center guidelines on the use of laboratory animals. The protocol for animal care and studies was approved by the Institutional Animal Care and Use Committee of SCMC (SCMC‐LAWEC‐2023‐006).

### Hemodynamic and Echocardiography Measurement

Right ventricular systolic pressure (RVSP) was blindly measured with a Millar catheter transducer (ADInstruments, SPR‐671NR) in mice and with a PE50 catheter in rats (ADInstruments, PE9050). Briefly, both mice and rats were anesthetized with 1.25% tribromoethanol (200 mg kg^−1^ per body weight for mice and 125 mg kg^−1^ per body weight for rats, i.p.). The Millar catheter was inserted into the RV of the mice after opening the chest. A PE50 catheter was used to measure RVSP in rats. The pressure signal was acquired by the PowerLab system (ADInstruments) and analyzed by LabChart8 software (ADInstruments).

Transthoracic echocardiography was performed on a VisualSonics Vevo 3100 ultrasound machine (FujiFilm VisualSonics Inc) using an MS550D (40 MHz) transducer. RV function was evaluated by tricuspid annular plane systolic excursion (TAPSE), and right ventricle free wall thickness (RVFWT) was also obtained.

### Immunofluorescent Staining and Histological Staining

Heart sections were fixed with 4% paraformaldehyde. For immunofluorescent staining, the sections from human or mouse heart were blocked with 0.1% Triton X‐100 and 5% donkey serum at room temperature for 1 h. After three washes, they were incubated with anti‐AXL (1:500, Invitrogen, Cat# PA5‐106118) and anti‐Vimentin (1:200, Invitrogen, Cat#MA5‐11883) at 4 °C overnight, followed by incubation with Alexa 594 conjugated anti‐rabbit IgG (1:1000, Invitrogen, Cat#A‐11012) and Alexa 488 conjugated anti‐mouse IgG (1:1000, Invitrogen, Cat#A‐11001) at room temperature for 1 h. Nuclei were counterstained with DAPI (Beyotime, Cat#P0131). Quantification of AXL expression was performed using ImageJ.

For immunohistochemistry staining, mouse heart sections were dewaxed and dehydrated. Antigen retrieval was performed with antigen retrieval buffer (Beyotime, Cat#P0085) for 5 min in a high‐pressure cooker. After blocking, slides were incubated with anti‐Collagen I (1:300, CST, Cat#72026T) at 4 °C overnight and were then incubated with HRP‐conjugated anti‐rabbit IgG at room temperature for 1 h. The cells were counterstained with hematoxylin. Collagen I^+^ areas were quantified blindly in 20 fields at 40× magnification per sample.

For the assessment of RV remodeling, paraffin sections of hearts were dewaxed and dehydrated, and then stained with the WGA staining kit (Invitrogen, Cat#W11261). Nuclei were counterstained with DAPI (Beyotime, Cat#P0131). The size of cardiomyocytes was quantified blindly in 20 fields at 40X magnification per sample. Paraffin‐embedded sections of heart were also dewaxed and dehydrated, and then stained with HE staining kit (Beyotime, Cat#C0105S) and Masson staining kit (Beyotime, Cat#C0189S) according to the manufacturers’ instructions.

### Primary Cultures of Mouse Cardiac Fibroblasts

Hearts were excised from 3‐day‐old neonatal mice and initially rinsed in a culture dish containing PBS. A digestion solution was prepared by dissolving 40 mg of Type II collagenase and 200 mg of bovine serum albumin (BSA) in 40 mL of PBS, followed by filtration. The hearts were then transferred to a vial containing digestion solution and agitated at 400 rpm on a magnetic stirrer set to 37 °C for 10 min. Subsequently, the digested supernatant was transferred to a centrifuge tube containing complete medium (DMEM/F12 medium supplemented with 10% FBS and 1% penicillin–streptomycin) and stored at 4 °C. The digestion process was repeated 6–8 times until the heart tissues were nearly completely digested. All supernatant collected was filtered through a 100 µm strainer and centrifuged at 1000 rpm for 5 min. After discarding the supernatant, the cell pellet was resuspended in complete medium and transferred to a 10 cm culture dish. The cells were incubated at 37 °C with 5% CO2 for 90 min to allow FB adhesion. Finally, the supernatant was discarded and replaced with fresh complete medium. Cells between passages 2 and 4 were used for subsequent experiments.

### siRNA Transfection and Adenovirus Infection

*Axl* siRNA, *Nfic* siRNA, and scramble siRNA were purchased from GenePharma Biotechnology and transfected into FBs at a concentration of 20 nm using RNAiMAX kit (Invitrogen, Cat#13778100) according to the manufacturer's instructions. Specifically, once the cell confluence reaches 40–50%, a transfection complex was formulated by blending DMEM medium, siRNAs, and Lipofectamine RNAiMAX reagent in suggested proportions according to the manufacturer's instructions.

This complex was then allowed to incubate for 5 min prior to its introduction into the cell culture medium. Adenovirus overexpressing *Axl* (AdAxl) and control adenovirus (AdCtrl) were purchased from Hanbio Biotechnology. FBs were infected with AdAxl or AdCtrl at a multiplicity of infection (MOI) of 50. The fluorescence of FBs was examined 48 h post‐infection to evaluate infection efficiency.

### Cell Proliferation and Migration

To assess FB proliferation after gene silencing or overexpression, 5×10^3^ FBs were seeded on a 96‐well plate to reach 40–50% confluency before gene manipulation, and then transfected with *Axl* siRNA or *Nfic* siRNA (20 nm) to knock down the target gene or with scramble siRNA as controls. FBs were infected with AdAxl to overexpress AXL or with AdCtrl as controls. 24 h post‐transfection or infection, FBs were starved for 24 h before further cultured either in a 37 °C incubator or in a hypoxia working station with 1%O_2_ for another 24 h. To explore whether *Nfic* silencing could restore the proliferation induced by *Axl* overexpression, FBs were first infected with AdAxl or AdCtrl for 48 h, followed by transfection of *Nfic* siRNA or scramble siRNA for 48 h. Cells were cultured at 37 °C incubator or in a hypoxia working station with 1%O_2_ for 24 h. To assess FB proliferation after inhibitor treatment, FBs were administered with R428 (1 µm), LY294002 (10 µm), KC7F2 (40 µm), or TC‐S 7009 (10 µm) and cultured at 37 °C incubator or in a hypoxia working station with 1%O_2_ for 24 h. Cells were fixed and immunostained with Click‐iT EdU‐488 kit (Servicebio, Cat#G1601). Nuclei were counterstained with DAPI. Images were taken using THUNDER microscopy (Leica) at 20× magnification for quantification.

To assess FB migration following gene silencing or overexpression, 1×10^6^ FBs were seeded on a 6‐well plate to reach 40–50% confluency prior to gene manipulation. Gene knockdown was achieved using siRNA targeting the specific gene, while overexpression was induced using adenovirus vectors. Appropriate controls were included as described above. Once the cells reached 100% confluency, they were starved for 24 h in preparation for the scratch assay. In brief, a sterile pipette tip was utilized to gently create uniform scratches across the cell monolayer in each well. After scratching, all wells were rinsed with PBS to remove detached cells. Subsequently, the culture medium was added to each well, and the plate was then incubated at 37 °C with 5% CO_2_ or within a hypoxia working station for 24 h. For assessing FB migration after inhibitor administration, cell monolayers were similarly scratched and rinsed with PBS. Subsequently, R428, LY294002, KC7F2, or TC‐S 7009 was added along with culture medium. Photographs of the scratched areas were taken to monitor cell migration.

### Single‐Nucleus RNA Sequencing (SnRNA‐seq) Analysis

RV tissues were harvested from HH‐induced PH mice, MCT‐administered PH rats, SuHx‐induced PH mice, and their corresponding control RV tissues. Frozen RV tissues from three rodents of the PH group or corresponding controls were dissociated into a single‐nucleus suspension (In brief, frozen tissue was crushed with a prechilled pestle in lysis buffer to form a homogeneous mixture. The homogenate was then passed through a centrifuge column to isolate nuclei from cellular debris. Subsequent purification steps involved debris removal solution and buffer washes. Finally, nuclei were resuspended in the recommended resuspension buffer, and nuclear integrity was assessed before proceeding.) and loaded on a 10× Genomics Chromium instrument to generate barcoded single nuclei for the construction of single‐nucleus cDNA libraries. SnRNA‐seq was performed on a HiSeq 4000 with pair‐end 150bp. SnRNA‐seq data were prefiltered to remove cells with a negative hashing tag or doublets using the R package ‘Seurat’.^[^ ^34^, ^35^, ^36^ ^]^ Briefly, nuclei that expressed fewer than 100 genes, nuclei that expressed over 4,000 genes, and nuclei with unique molecular identifiers (UMIs) more than 10% from the mitochondrial genome were filtered out. The data were normalized and integrated in Seurat, followed by Scaled and summarizing by principal component analysis (PCA), and then visualized using a UMAP plot. The FindClusters function in Seurat was used to cluster cells based on the gene expression profile with a resolution of 0.4. The CellMarker (http://xteam.xbio.top/CellMarker/) database was used to annotate cells.^[^ ^37^ ^]^ RV FBs transcriptomes were extracted for differential gene expression, FBs subpopulation clusters, pseudotime, and CellChat prediction.^[^ ^38^, ^39^, ^40^ ^]^ For RV FBs subpopulation clustering, FB subpopulation was defined according to their *Axl* expression level. To infer the gene expression changes of FBs during the development of PH disease, pseudotime analysis was used. To predict the ligand‐receptor interaction, the CellChat pipeline was used.

### Real‐Time Polymerase Chain Reaction (RT‐PCR)

Total RNA was isolated from murine RV tissues or FBs using RNA easy animal extraction kit (Beyotime, Cat#R0027). One microgram of RNA was transcribed into cDNA using the HiScript IV All‐in‐One Ultra RT SuperMix for qPCR (Vazyme, Cat#R433‐01) according to the manufacturer's protocol. Quantitative RT‐PCR analysis was performed on a QuantStudio 3 system (Applied Biosystems) with the ChamQ SYBR Color qPCR Master Mix (Vazyme, Cat#Q411‐02). Target mRNA was determined using the comparative cycle threshold method of relative quantitation. *Gapdh* was used as an internal control for the analysis of the expression of mouse genes. The primer sequences were provided in Table S2 (Supporting Information).

### Western Blot

Protein lysates were prepared by mincing RV tissues from mice or harvesting cells in cold RIPA lysis buffer supplemented with protease inhibitor cocktails. For the separation of nuclear and cytoplasmic proteins, the collected cells were processed using a NE‐PER nuclear‐cytoplasmic extraction kit (Thermo Scientific, Cat# 78833) according to the instructions provided with the kit. An equal amount of protein was loaded for SDS‐PAGE. The PVDF membranes were blotted with anti‐AXL (1:1000, Invitrogen, Cat# PA5‐106118), anti‐NFIC (1:1000, CST, Cat# 11911), anti‐AKT (1:1000, abmart, Cat# T55561), anti‐p‐AKT (1:1000, abmart, Cat# T40067) or anti‐GAPDH (1:2000, abmart, Cat# P30008) antibodies. HRP‐Goat‐anti‐Rabbit IgG (1:8000, Beyotime, Cat# A0208) was used as the second antibody. The quantification of densitometric measurements for blot images was performed on ImageJ.

### Chromatin Immunoprecipitation (ChIP)‐PCR

The ChIP experiment was conducted using the SimpleChIPPlus Enzymatic Chromatin IP Kit (CST, Cat# 9005). First, FBs were plated at a density of 5*10^6 cells per 15 cm dish. Cells were either infected with AdCtrl or AdAxl at an MOI of 50. After adenovirus infection for 36 h, the cells were starved for 18 h, followed by culture in the incubator (normoxic condition) or hypoxic working station at 37 °C for 24 h. Subsequently, the ChIP procedure was initiated. The cells were fixed with formaldehyde and quenched with glycine. The cells were then collected, washed, and resuspended in buffer for nuclear preparation and chromatin digestion. Micrococcal nuclease was used to digest the DNA at 37 °C for 30 min, and the reaction was terminated with EDTA. The anti‐NFIC antibody (CST, Cat# 11911) was then added and incubated at 4 °C for 36 h, followed by the addition of beads for an additional 2.5 h of incubation. The target protein‐DNA‐beads complex was precipitated using immunoprecipitation. Afterward, multiple washes were performed to remove nonspecific binding. The bound chromatin was then eluted from the antibody/beads and subjected to crosslink reversal. Finally, the DNA samples were purified through a centrifugal column for subsequent PCR analysis.

PCR was performed utilizing TaKaRa Ex Taq Hot Start Version (Takara, Cat# RR006A). The procedure involved preparing a reaction mixture containing TaKaRa Ex Taq HS enzyme, 10× Ex Taq buffer, and dNTP mixture. Purified DNA template and customized primers were then introduced into this mixture. In the PCR instrument, the denaturation step was performed at 98 °C for 10 s, followed by annealing at 55 °C for 30 s and extension at 72 °C for 1 min, with these steps being cycled 30 times. Finally, the amplified products were detected and analyzed using DNA electrophoresis. The primer sequences used for ChIP‐PCR are listed below: Forward primer: AGCTGTTATTTATTAGAAAGGTG; Reverse primer: ACTAAGAGTCAGATATGTGGT.

### ELISA

Collagens were measured in cell lysates using COL1A1 ELISA kit (biotechwell, Cat# EM30923S), COL3A1 ELISA kit (biotechwell, Cat# EM30924S), COL8A1 ELISA kit (biotechwell, Cat# EM30930S), COL4A1 ELISA kit (biotechwell, Cat# EM30926S), COL4A2 ELISA kit (biotechwell, Cat# EM30927S), COL5A1 ELISA kit (biotechwell, Cat# EM30928S), and COL5A2 ELISA kit (biotechwell, Cat# EM30929S). All experiments were conducted according to the manufacturer's instructions. A microplate reader (TECAN, Infinite 200 PRO) was used to measure absorbance at 450 nm. A standard curve was generated to convert absorbance to protein concentration.

### Data Availability

The snRNA‐seq data that support the findings of this study are available from the corresponding author upon reasonable request. The bulk transcriptome data supporting this study have been deposited in the Gene Expression Omnibus (GEO) database under the accession numbers GSE198618, GSE240921, GSE240923, and GSE242014.^[^ ^41^, ^42^ ^]^

### Statistical Analysis

Statistical analysis was performed with Prism 9 (Graphpad Software Inc.). The Kolmogorov–Smirnov test was used to assess the normality of data. Two‐group comparisons were compared by the unpaired 2‐tailed Student t test for equal variance or the Mann–Whitney U test for unequal variance. Multiple comparisons were performed by one‐way ANOVA with a Tukey post hoc analysis that calculates corrected P values. The Kruskal–Wallis test was used for multiple comparisons that do not follow a normal distribution. *P* less than 0.05 indicated a statistically significant difference. All Data are presented as mean ± SEM.

### Ethics Approval and Consent to Participate

This study has been approved by the Ethics Committee of Shanghai Children's Medical Center, affiliated to Shanghai Jiao Tong University School of Medicine (SCMCIRB‐K2024081‐1), and informed consent has been obtained from the patients or their relative guardians.

## Conflict of Interest

The authors declare no conflict of interest.

## Author Contributions

L.‐W. W., M.C., and C.‐Y.J. contributed equally to this work and shared first authorship. Y.Y. and H.Z. contributed equally to this work and shared last authorship. L.‐W.W., M.C., and C.‐Y.J. carried out animal experiments, contributed to data acquisition, analysis, and drafted the manuscript. D.‐J.J., X.Z., and X.‐H.X. carried out animal experiments, histological staining, and flow cytometry. Y.‐W.L., B.F., L.‐C.Y., and Y.‐Y.H. contributed to data interpretation and manuscript revision. X.H. contributed to the organ harvest and Western blot. Y.‐C.Z. performed RT‐PCR for gene validation. X.‐L.Z., Y.S., and T.‐Y.L. contributed to data interpretation. L.‐J.F. provided intellectual input and funding support. Y.Y. and H.Z. conceived and supervised the study, provided funding support, and revised the manuscript.

## Supporting information



Supporting Information

## Data Availability

The snRNA‐seq data in support of this study are available from the corresponding author upon reasonable request. The bulk transcriptome data supporting this study were deposited in the Gene Expression Omnibus (GEO) database under accession numbers GSE198618, GSE240921, GSE240923, and GSE242014.
