# Supplementary material for: Inactivation of AXL in Cardiac Fibroblasts Alleviates Right Ventricular Remodeling in Pulmonary Hypertension
Source: Adv Sci (Weinh). 2025 Dec 3;13(9):e08995. doi: 10.1002/advs.202508995 (PMC12903973; doi:10.1002/advs.202508995)
Supplement: Supplementary file 1 — Supporting Information [file ADVS-13-e08995-s001.docx]

**Inactivation of AXL in Cardiac Fibroblasts Alleviates Right Ventricular Remodeling in Pulmonary Hypertension**

Li-Wei Wu^1,2,#^, Min Chen^3,#^, Chen-Yu Jiang^1,2,#^, Dai-Ji Jiang^1,2^, Xu Zhang^1^, Xiao-He Xu^4^, Yi-Wei Liu^1,2,5^, Bei Feng^1,5^, Lin-Cai Ye^1,5^, Yang-Yang He^4^, Xu Huang^1,2^, Yi-Chi Zhang^1,2^, Xing-Liang Zhou^1,2^, Yi Shen^1,2^, Tian-Yu Liu^1,2^, Li-Jun Fu^1,6^, Yi Yan^1,5,^*, Hao Zhang^1-3,5,^*

1. Heart Center and Shanghai Institute of Pediatric Congenital Heart Disease, Shanghai Children’s Medical Center, National Children’s Medical Center, Shanghai Jiao Tong University School of Medicine, Shanghai, China
2. Department of Cardiothoracic Surgery, Shanghai Children's Medical Center, National Children's Medical Center, Shanghai Jiao Tong University School of Medicine, Shanghai, China.
3. Children’s Heart Center, Institute of Cardiovascular Development and Translational Medicine, The Second Affiliated Hospital and Yuying Children’s Hospital, Wenzhou Medical University, Wenzhou, China
4. School of Pharmacy, Henan University, Kaifeng, China
5. Shanghai Research Center for Pediatric Cardiovascular Diseases, Shanghai Children's Medical Center, National Children's Medical Center, Shanghai Jiao Tong University School of Medicine, Shanghai, China
6. Department of Cardiology, Shanghai Children's Medical Center, National Children's Medical Center, Shanghai Jiao Tong University School of Medicine, Shanghai, China

# These authors contributed equally to this work and shared first authorship

* These authors contributed equally to this work and shared last authorship

**Correspondence to:**

**Hao Zhang**, Heart Center and Shanghai Institute of Pediatric Congenital Heart Disease; Shanghai Clinical Research Center for Rare Pediatric Diseases; and Shanghai Research Center for Pediatric Cardiovascular Diseases, Shanghai Children’s Medical Center, Shanghai Jiao Tong University School of Medicine, Shanghai, China. Children’s Heart Center, Institute of Cardiovascular Development and Translational Medicine, The Second Affiliated Hospital and Yuying Children’s Hospital, Wenzhou Medical University, Wenzhou, China. E-mail: [drzhanghao@126.com](mailto:drzhanghao@126.com) (zhang-hao@scmc.com.cn)

**Yi Yan**, Heart Center and Shanghai Institute of Pediatric Congenital Heart Disease; Shanghai Clinical Research Center for Rare Pediatric Diseases; and Shanghai Research Center for Pediatric Cardiovascular Diseases, Shanghai Children’s Medical Center, Shanghai Jiao Tong University School of Medicine, Shanghai, China. E-mail: [yannie0928@163.com](mailto:yannie0928@163.com) (yanyi@scmc.com.cn)

**Table S1**. The clinical information of human subjects involved in this study

**Table S2.** The primers used in RT-PCR

**Figure S1.** Hemodynamics and RV structural assessment in mice under hypobaric hypoxia (HH) conditions

**Figure S2.** Assessment of RV fibrosis in PH

**Figure S3.** Representative marker genes for each cell type

**Figure S4.** Alteration of cell communications in RVs from PH mice

**Figure S5.** *Axl* expression pattern in cardiac fibroblasts (FBs) from RVs of PH

**Figure S6.** Trajectory analysis of FBs from RVs of PH mice in comparison with control mice

**Figure S7.** Expression levels of *AXL* during RV remodeling

**Figure S8.** The effect of AAV9-Postn-hAXL delivery on Axl expression in RVs or RV FBs of mice

**Figure S9.** AXL overexpression in cardiac FBs aggravates RV function after HH exposure

**Figure S10.** The efficiency of *Axl* knockdown in RVs or RV FBs of *Axl*^△FB^ mice

**Figure S11.** The impact of HH condition on scores associated with active phenotype in RV FBs

**Figure S12.** Purity of isolated primary mouse cardiac fibroblasts

**Figure S13.** The alteration of collagen content in cell lysates from primary mouse FBs after hypoxia exposure

**Figure S14.** The efficiency of Axl silencing in primary mouse cardiac FBs

**Figure S15.** The enrichment of modules according to protein-protein interactions of genes positively correlated with *Axl*

**Figure S16.** The effect of R428 on synthesis of collagens produced by primary mouse cardiac FBs infected with AdAxl or AdCtrl under hypoxic conditions

**Figure S17.** Expression patterns of *Axl* in the RVs from rats administrated with MCT

**Figure S18.** Temporal profiling of AXL expressions in RVs of MCT-induced PH rats

**Figure S19.** *Axl* overexpression in cardiac FBs aggravates RV remodeling in MCT-induced PH

**Figure S20.** Expression patterns of *Axl* in the RVs from mice after treatment with SU5416 and hypoxia

**Figure S21.** Temporal profiling of AXL expressions in RVs of suHx-induced PH mice

**Figure S22.** Genetic ablation of *Axl* in cardiac FBs attenuated RV remodeling after suHx challenge

**Figure S23.** AXL protein levels in the lungs of PH animal models

**Figure S24.** *Axl* overexpression in cardiac FBs aggravates RV remodeling in PAB mouse model

**Figure S25.** R428 treatment rescues RV remodeling in PAB mouse model

**Figure S26.** The effect of *Nfic* knockdown on the proliferation of FBs in response to hypoxia

**Figure S27.** The effect of *Nfic* knockdown on synthesis of collagen in FBs infected with AdAxl or AdCtrl under hypoxic conditions

**Figure S28.** The effect of PI3K inhibitor on synthesis of collagen in FBs infected with AdAxl or AdCtrl under hypoxic conditions

**Figure S29.** The effect of HIF1α inhibitor on phenotypes of FBs infected with AdAxl or AdCtrl under hypoxic conditions

**Figure S30.** The effect of HIF2α inhibitor on phenotypes of FBs infected with AdAxl or AdCtrl under hypoxic conditions

**Table S1**. **The clinical information of human subjects involved in this study**

|  | Con1 | Con2 | Con3 | PAH1 | PAH2 | PAH3 |
| --- | --- | --- | --- | --- | --- | --- |
| Gender | F | F | M | M | F | F |
| Age (y) | 7 | 6 | 13 | 6.0 | 13.0 | 11.0 |
| 6MWD (m) | / | / | / | 333.05 | 294.88 | 282 |
| WHO FC | / | / | / | III | IV | IV |
| NT-proBNP (pg/ml) | / | / | / | 2445 | 1286 | 4311 |
| mRAP (mmHg) | / | / | / | 14 | 10 | / |
| CI (L/min/m^2^) | / | / | / | 3.89 | 2.65 | / |
| mPAP (mmHg） | / | / | / | 80 | 77 | / |
| PVRi (WU x m^2^) | / | / | / | 25.96 | 19.33 | / |
| RV/LV diameter | / | / | / | 1.82 | 1.04 | 2.33 |
| PAAT (ms) | / | / | / | 81 | 106 | 92 |
| RVFAC (%) | / | / | / | 15.6 | 21.58 | 18 |
| TAPSE (cm) | / | / | / | 1.32 | 1.38 | 0.87 |
| TRV (m/s) | / | / | / | 5.94 | 4.76 | 6.02 |
| RVEDVI (mL/m^2^) | / | / | / | 153.67 | 145.96 | 277.07 |
| RVESVI (mL/m2) | / | / | / | 103.17 | 68.3 | 219.97 |
| RVEF (%) | / | / | / | 32.86 | 31.1 | 20.61 |
| Targeted drugs | / | / | / | Bosentan+Tadalafil+ Treprostinil | bosentan+sidenafil+  Treprostinil | bosentan+  tadalafil+  Treprostinil |

6MWD: six minute walking distance; WHO FC: World Health Organization functional class; NT-proBNP: N-Terminal pro-B-type natriuretic peptide; mRAP: mean right atrial pressure; CI: cardiac index; mPAP: mean pulmonary arterial pressure; PVRi: pulmonary vascular resistance index; PAAT: pulmonary artery acceleration time; RVFAC: right ventricular fractional area change; TAPSE: tricuspid annular plane systolic excursion; TRV: tricuspid regurgitation velocity; RVEDVI: right ventricular end-diastolic volume index; RVESVI: right ventricular end-systolic volume index; RVEF: right ventricular ejection fraction.

**Table S2**. **The primers used in RT-PCR**

| Gene | Species | Forward primer sequence | Reverse primer sequence |
| --- | --- | --- | --- |
| *Axl* | *Mus musculus* | GACAACCCGGCCCTGC | CCTCGGTCTGTGTGTCCTTA |
| *Nppa* | *Mus musculus* | GGGCTCCTTCTCCATCACC | GACCTCATCTTCTACCGGCAT |
| *Nppb* | *Mus musculus* | GAGTCCTTCGGTCTCAAGGC | ACTTCAGTGCGTTACAGCCC |
| *Gapdh* | *Mus musculus* | TCAACGACCCCTTCATTGACC | CACCAGTAGACTCCACGACA |
| *Nppa* | *Rattus norvegicus* | GCTTCCAGGCCCTGTGTG | GGAGGTTCAGAGACGGGCAT |
| *Nppb* | *Rattus norvegicus* | TGCTGGTGCCTTTGGGGAGT | CGCTTACTTGGCCCCCCGTC |
| *Gapdh* | *Rattus norvegicus* | TGAACGGGAAGCTCACTGG | TCCACCACCCTGTTGCTGTA |


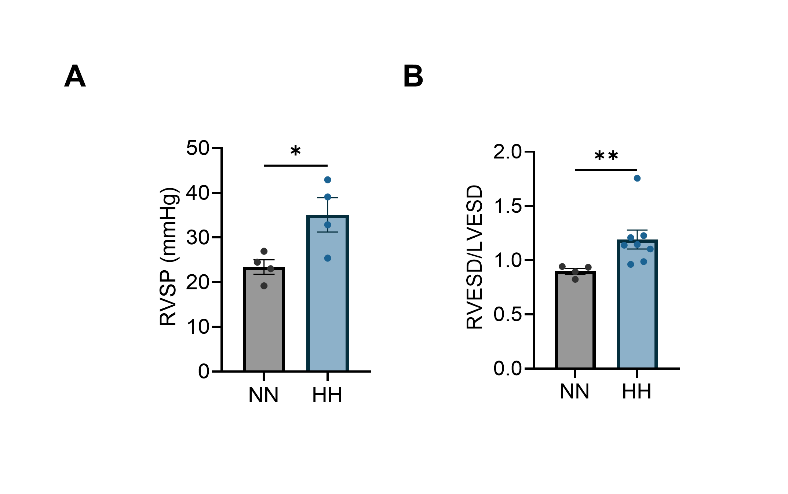


**Figure S1. Hemodynamics and RV structural assessment in mice under hypobaric hypoxia (HH) conditions**. **(A)** Right ventricular systolic pressure (RVSP) was measured by right heart catheterization in mice under hypobaric hypoxia (HH) conditions for 4 weeks or in ambient air (NN); n=4/group. **(B)** RV enlargement indicated by RVEDD/LVEDD was observed in mice under HH conditions (n=4-8/group). Data represent mean ± SEM. **P* < 0.05, ***P* < 0.01 compared to controls, as analyzed by unpaired *t* test or Mann-Whitney *U* test as appropriate.


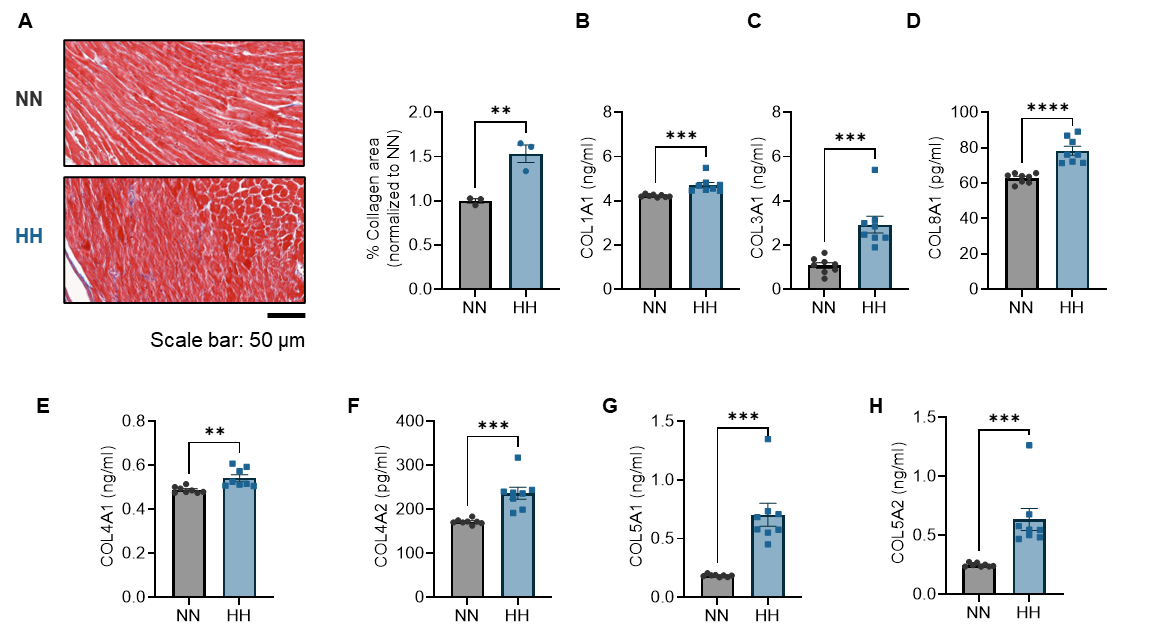


**Figure S2. Assessment of RV fibrosis in PH.** **(A)** Representative images of Masson staining and quantification of the collagen deposited in RVs from PH mice and control mice (n=3/group); scale bar = 50 μm. **(B-H)** The protein level (assessed by ELISA) of COL1A1 **(B)**, COL3A1 **(C)**, COL8A1 **(D)**, COL4A1 **(E)**, COL4A2 **(F)**, COL5A1 **(G)** and COL5A2 **(H)** in RVs from PH and control mice (n=8/group). Data represent mean ± SEM. ***P* < 0.01, ****P* < 0.001, *****P* < 0.0001 compared to controls, as analyzed by unpaired *t* test or Mann-Whitney *U* test as appropriate.


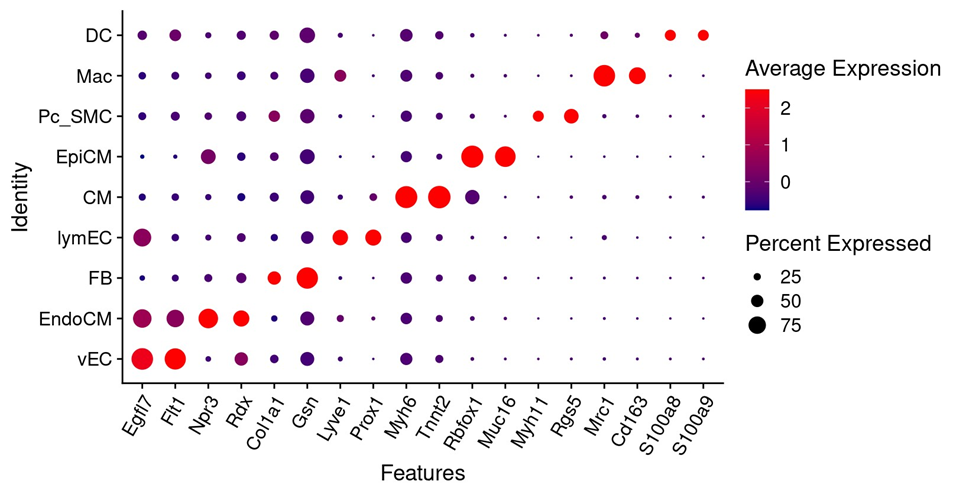


**Figure S3. Representative marker genes for each cell type.** The marker genes of each cell cluster in RVs were visualized in dot plot. The size of the dot represented the percentage of cells expressing the indicated gene. Red indicated high expression, and blue indicated low expression.


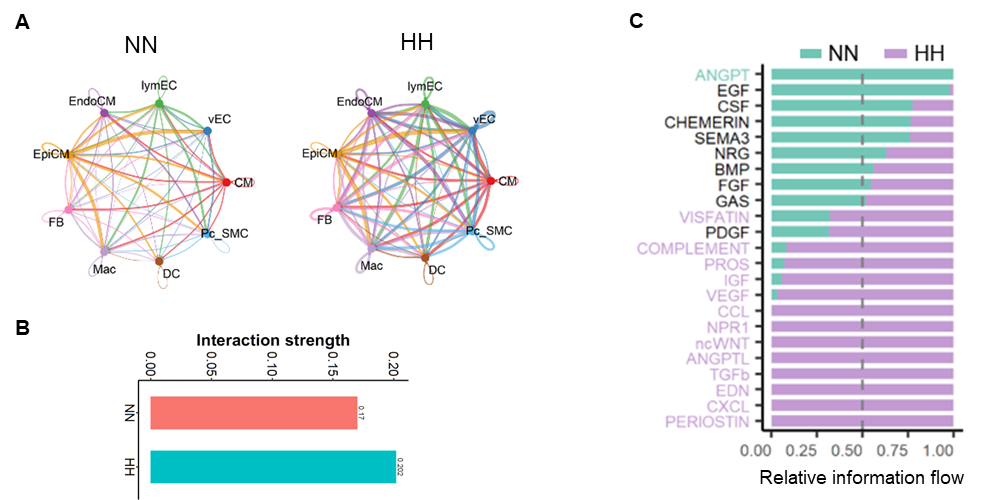


**Figure S4. Alteration of cell communications in RVs from PH mice.** **(A)** Cell communication strength between each cell type in RVs from PH mice and control mice. **(B)** All cell communication strength in RVs was compared between PH and control mice. **(C)** Enrichment of cell communication pathways in RVs from PH mice versus those in control mice.


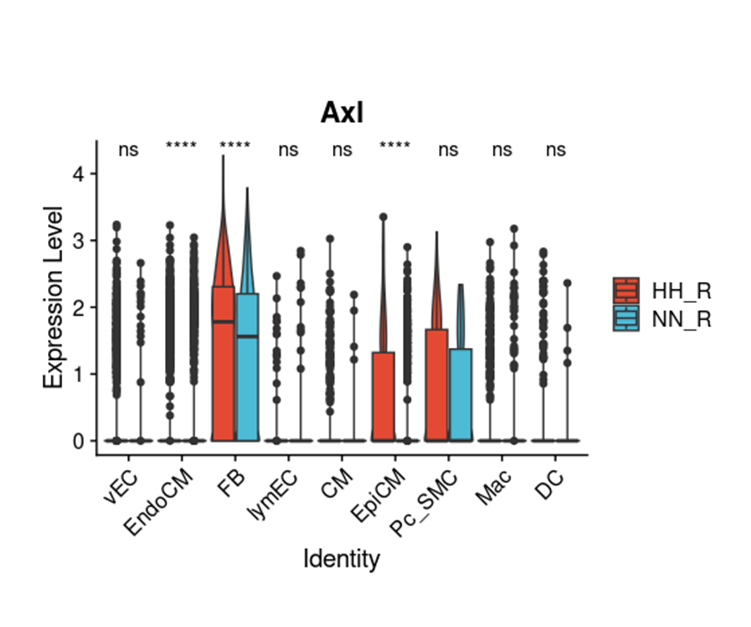


**Figure S5. *Axl* expression pattern in cardiac fibroblasts (FBs) from RVs of PH.** The expression levels of *Axl* in RV FBs from PH and control mice. *****P* < 0.0001 compared to indicated group, as analyzed by Wilcoxon test.


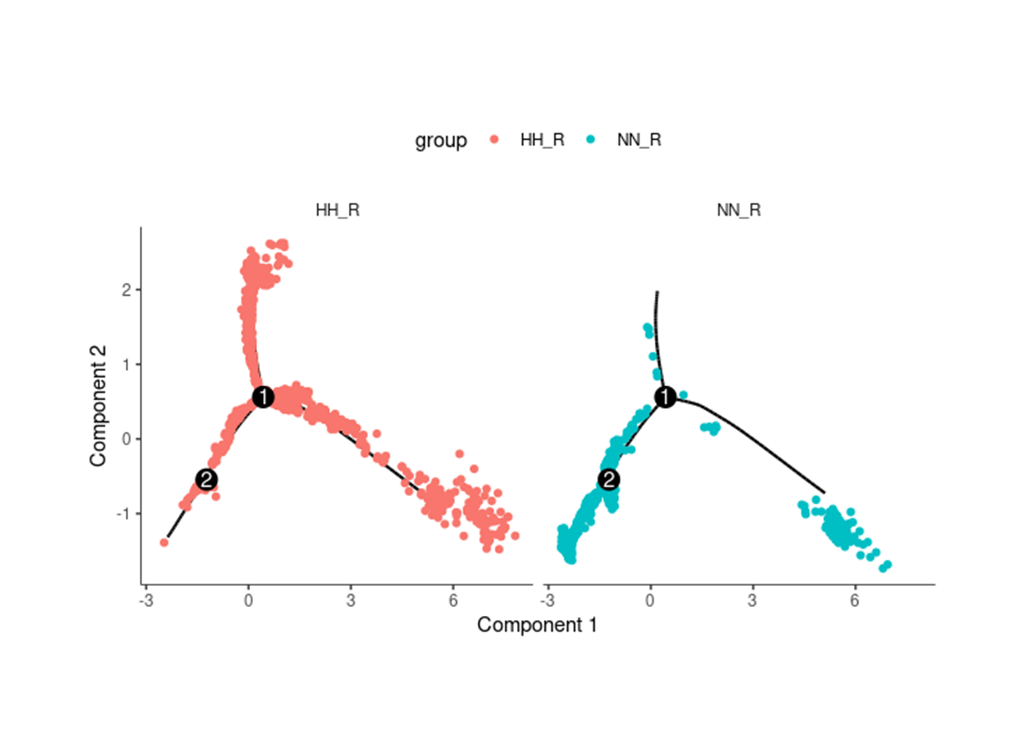


**Figure S6. Trajectory analysis of FBs from RVs of PH mice in comparison with control mice.** The trajectory of FBs from RVs were visualized in PH mice (left panel in red) and control mice (right panel in green).


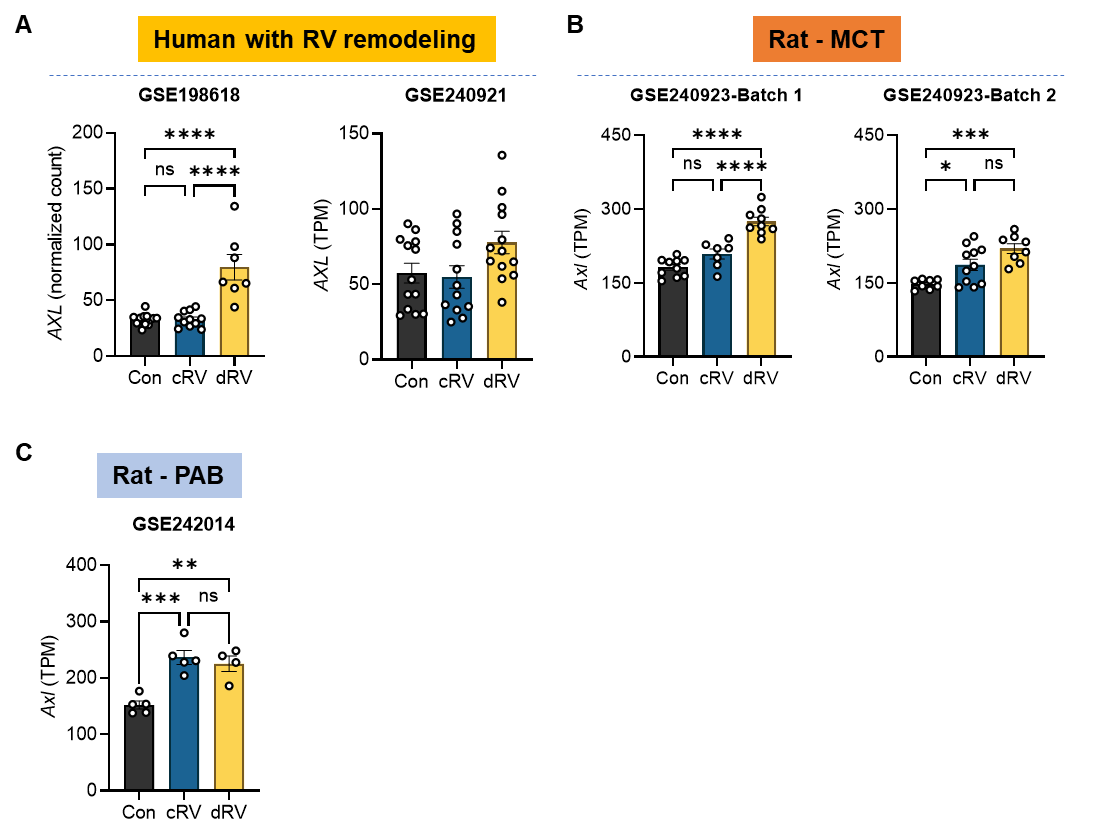


**Figure S7.** **Expression levels of *AXL* during RV remodeling.** **(A)** Expression levels of *AXL* in RVs from control subjects and PH patients with compensated or decompensated cardiac states from dataset GSE198618 (left panel; n = 7-14/group) and dataset GSE240921 (right panel; n=12-14/group). **(B)** Expression levels of *Axl* in RVs from control rats and MCT rats with compensated or decompensated cardiac states from dataset GSE240923 in first batch (left panel; n=7-10/group) and second batch (right panel; n=8-11/group). **(C)** Expression levels of *Axl* in RVs from control rats and PAB rats with compensated or decompensated cardiac states from dataset GSE242014. Data represent mean ± SEM. * *P* < 0.05, ** *P* < 0.01, *** *P* < 0.001, *****P* < 0.0001 compared to indicated group, as analyzed by One-way ANOVA test or Kruskal-Wallis test as appropriate.


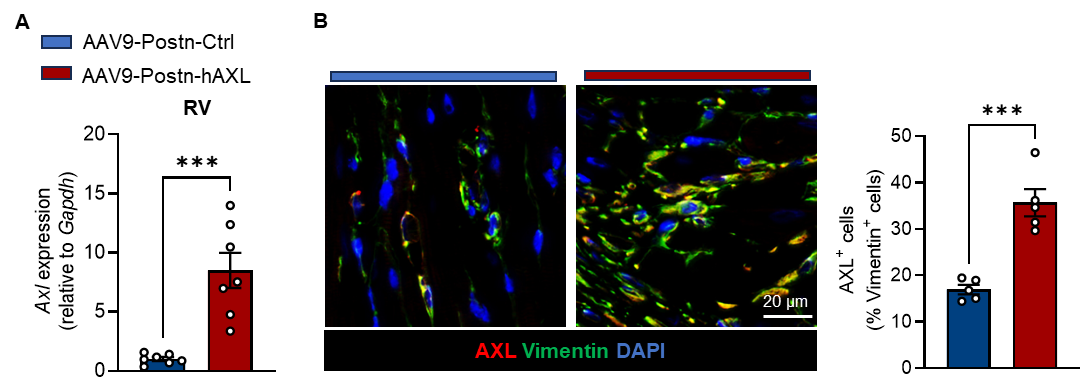


**Figure S8. The effect of AAV9-Postn-hAXL delivery on *Axl* expression in RVs or RV FBs of mice. (A)** Expression of *Axl* at mRNA level in RV tissues from mice receiving AAV9-Postn-hAXL or AAV9-Postn-Ctrl under normoxic condition (n=7/group). **(B)** Representative images of double immunofluorescent staining against AXL and Vimentin (left panel) and quantification (right panel) of AXL in RV FBs from mice receiving AAV9-Postn-hAXL or AAV9-Postn-Ctrl under normoxic condition (n=5/group). Data represent mean ± SEM. ****P* < 0.001 compared to AAV9-Postn-Ctrl recipients, as analyzed by unpaired *t* test. Scale bar: 20 μm.


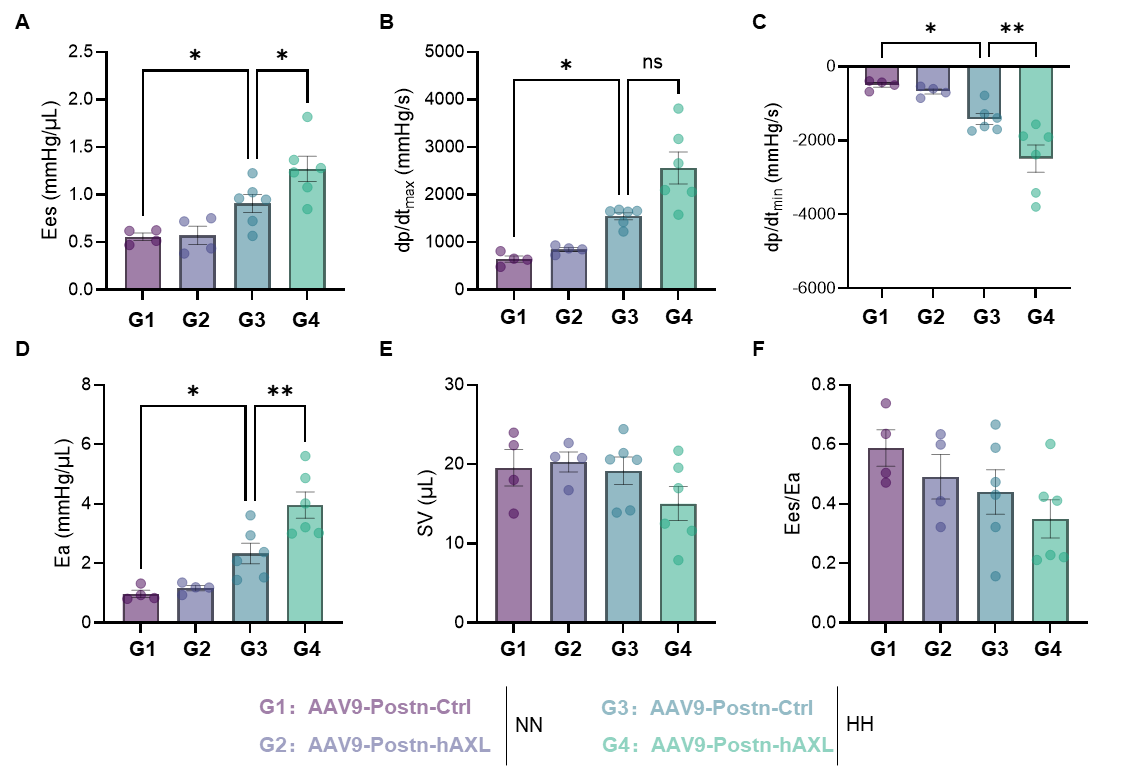


**Figure S9. AXL overexpression in cardiac FBs aggravates RV function after HH exposure.** **(A-F)** End-systolic elastance (Ees; n=4-6/group) **(A)**; dp/dt_max_ (n=4-6/group) **(B)**; dp/dt_min_ (n=4-6/group) **(C)**; arterial elastance (Ea; n=4-6/group) **(D)**; stroke volume (SV; n=4-6/group) **(E)** and coupling efficiency (Ees/Ea) **(F)** in PH or control mice receiving AAV9-Postn-hAXL or AAV9-Postn-Ctrl. Data are presented as mean ± SEM. * *P* < 0.05, ** *P* < 0.01 compared to indicated group, as analyzed by One-way ANOVA test or Kruskal Wallis as appropriate.


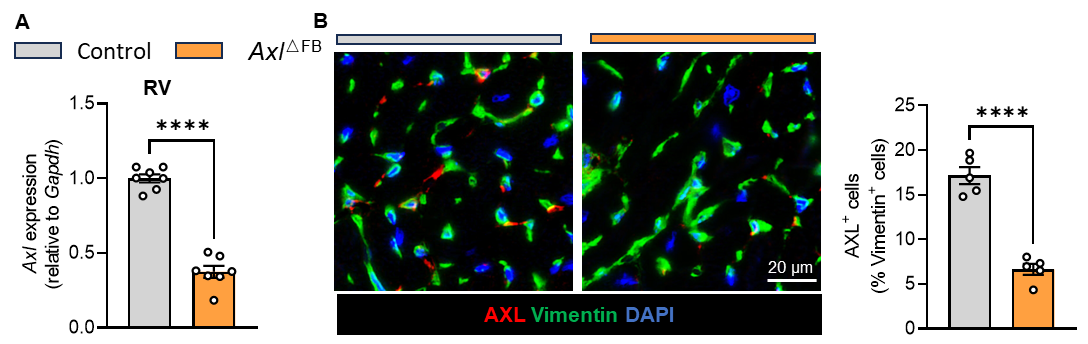


**Figure S10. The efficiency of *Axl* knockdown in RVs or RV FBs of *Axl*^△FB^ mice**. **(A)** Expression of *Axl* at mRNA level in RVs from *Axl^flox/flox^* mice receiving AAV9-Postn-Cre (*Axl*^△FB^) or AAV9-Postn-Ctrl (Control); n=7/group. **(B)** Representative images of double immunofluorescent staining against AXL and Vimentin (left panel) and quantification (right panel) of AXL in RV FBs from *Axl*^△FB^ or control mice (n=5/group). Data represent mean ± SEM. *****P* < 0.0001 compared to controls, as analyzed by unpaired *t* test. Scale bar: 20 μm.


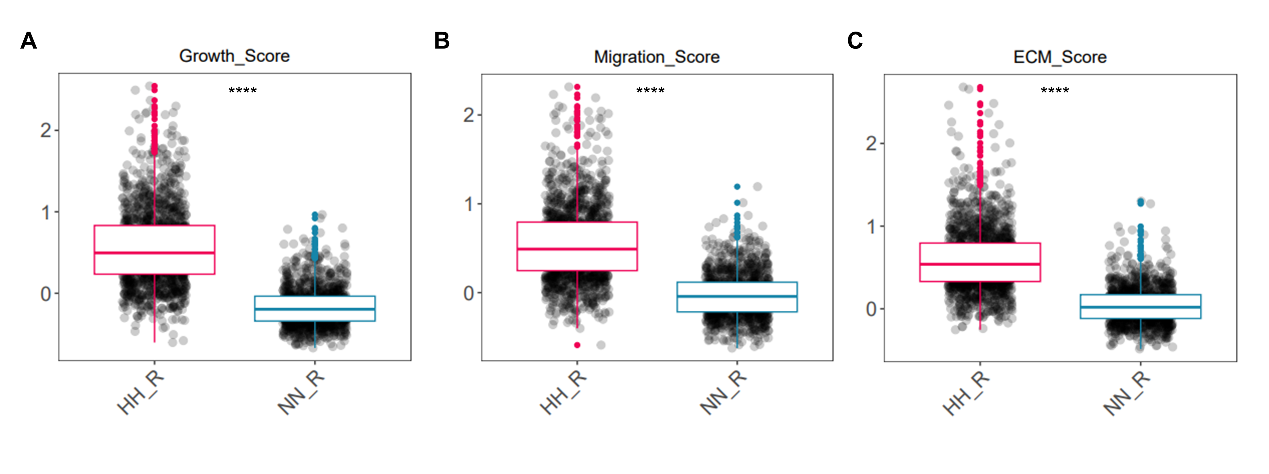


**Figure S11.** **The impact of HH condition on scores associated with active phenotype in RV FBs**. **(A-C)** Growth score **(A)**, Migration score **(B)** and ECM score **(C)** of RV FBs from mice under HH conditions and in ambient air. Each dot represents an individual FB.


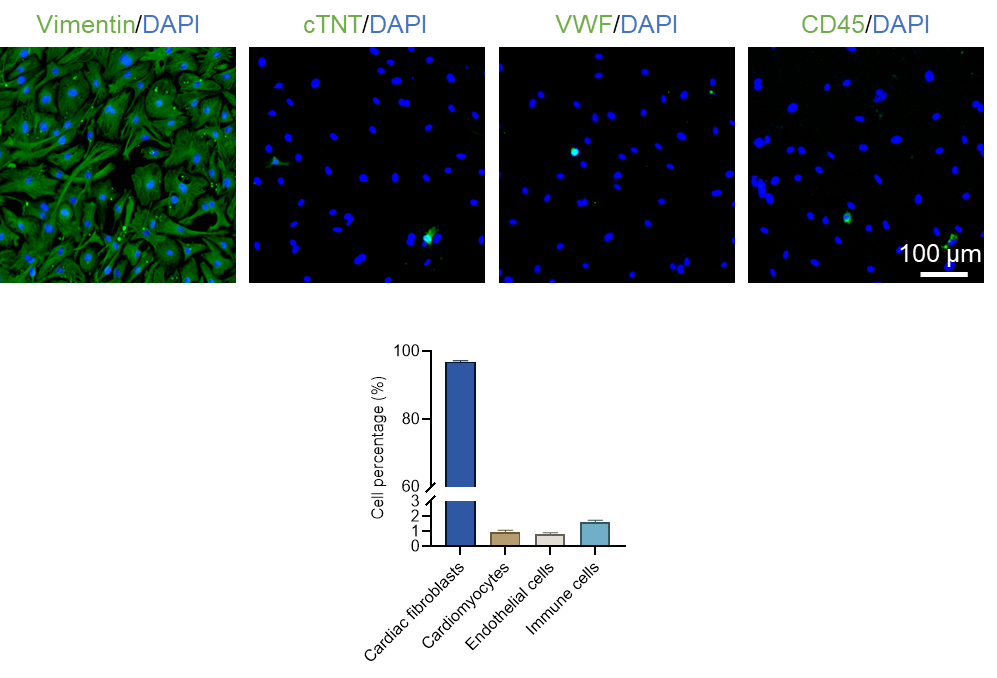


**Figure S12. Purity of isolated primary mouse cardiac fibroblasts.** Representative images of immunofluorescence staining for cell markers (Vimentin for fibroblasts, cTNT for cardiomyocytes, VWF for endothelial cells, and CD45 for immune cells) and quantification of each cell type in cultured primary mouse cardiac fibroblasts. Data are presented as mean ± SEM.


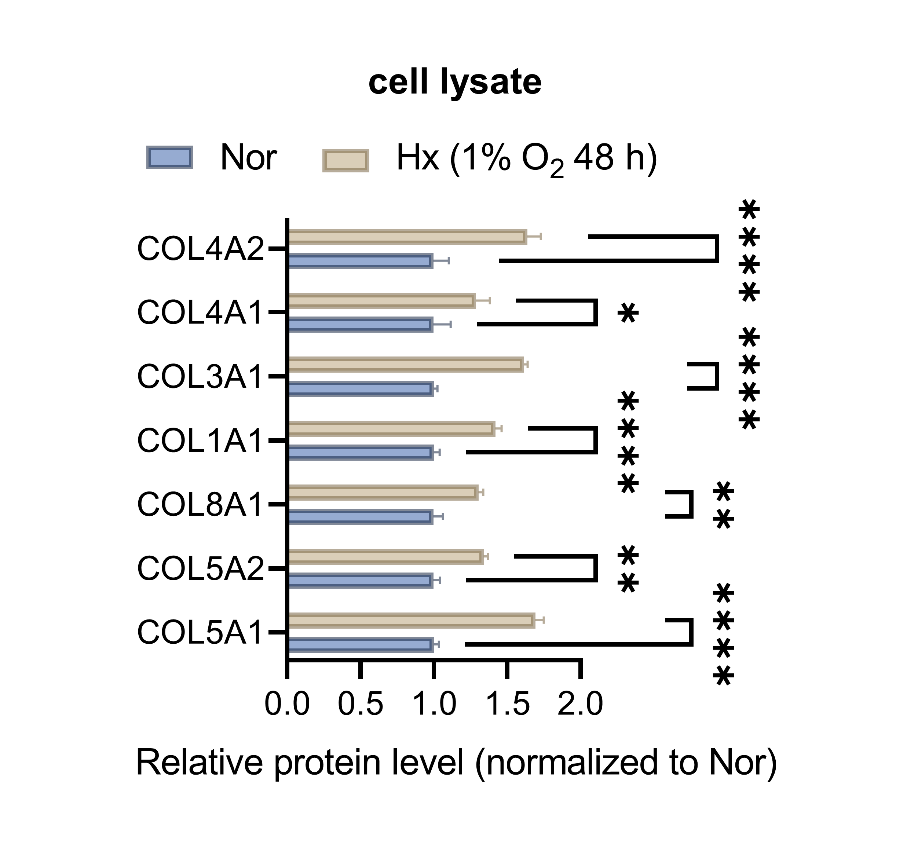


**Figure S13. The alteration of collagen content in cell lysates from primary mouse FBs after hypoxia exposure.** The collagen content was measured by ELISA in cell lysates from primary mouse FBs exposed to hypoxia (1%O_2_) or normoxic conditions for 48 h. Data represent mean ± SEM. **P* < 0.05, ***P* < 0.01, *****P* < 0.0001 compared to FBs under normoxic conditions, as analyzed by unpaired *t* test.


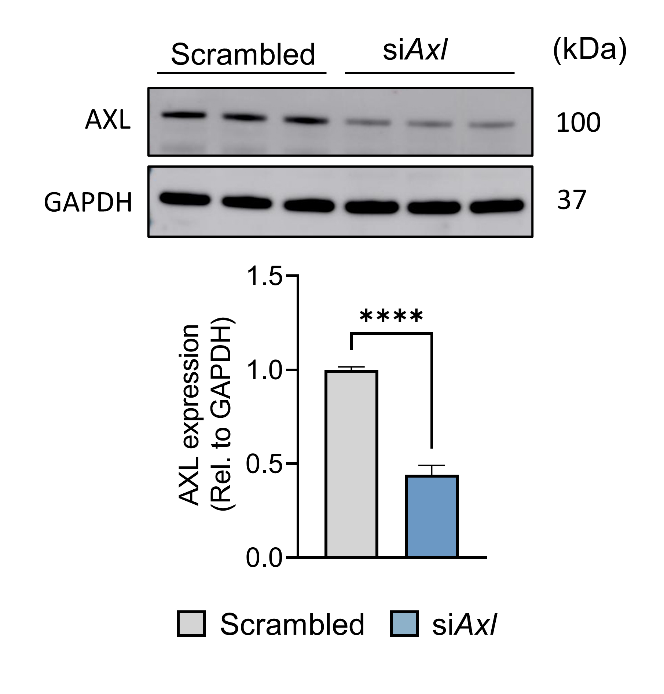


**Figure S14. The efficiency of Axl silencing in primary mouse cardiac FBs.** AXL protein level was reduced in si*Axl*-transfected primary cardiac FBs compared to scrambled siRNA infected cells (all distributed in two independent experiments). Data represent mean ± SEM. *****P* < 0.0001 compared to indicated group, as analyzed by unpaired *t* test.


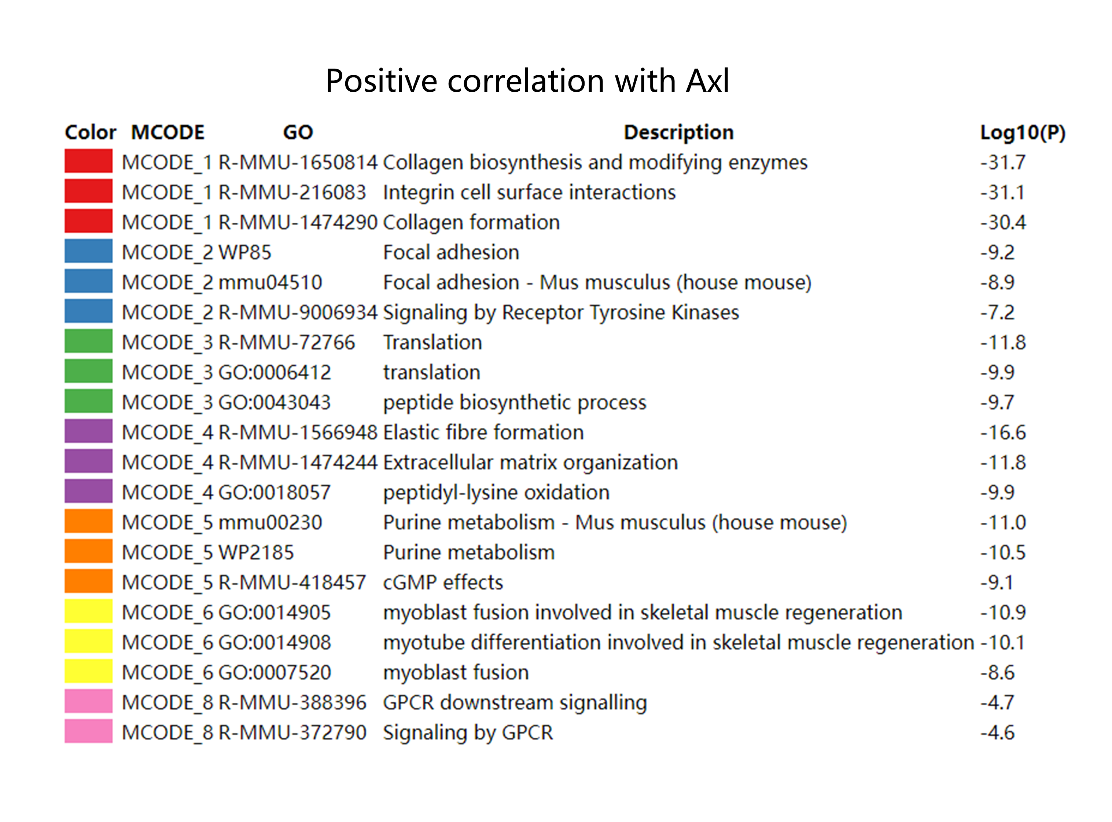


**Figure S15. The enrichment of modules according to protein-protein interactions of genes positively correlated with *Axl*.** Genes in positive correlation with *Axl* expression were sorted and analyzed by protein-protein interactions analysis. Modules were generated using MCODE and represented in different colors. The enriched pathways of genes within each module were depicted.


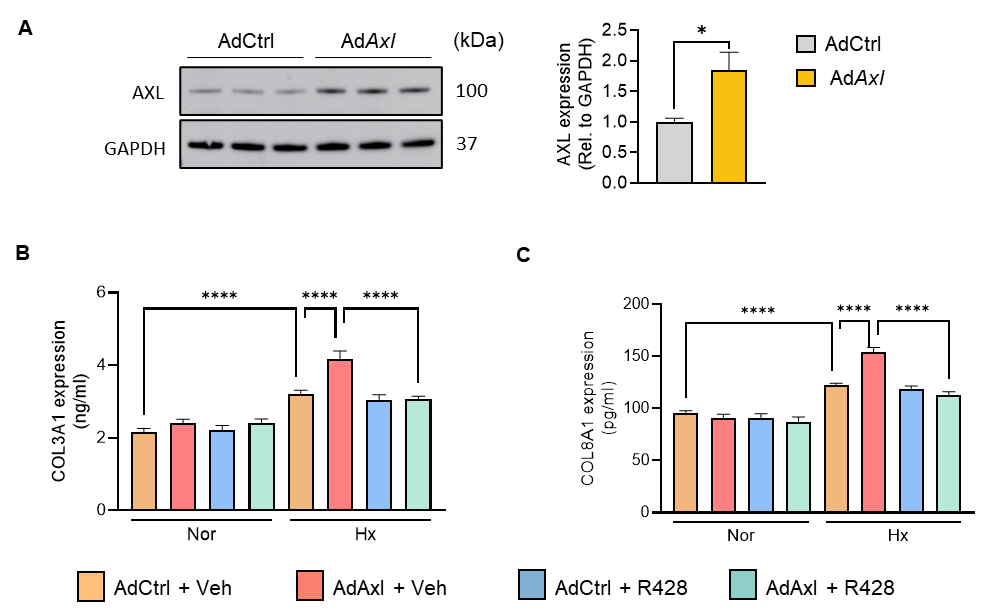


**Figure S16. The effect of R428 on synthesis of collagens produced by primary mouse cardiac FBs infected with AdAxl or AdCtrl under hypoxic conditions.** **(A)** AXL protein level was increased in Ad*Axl* infected cardiac FBs compared to AdCtrl infected cells (all distributed in two independent experiments). **(B-C)** R428 reduced the protein levels of COL3A1 **(B)** and COL8A1 **(C)** in cell lysates from primary mouse FBs in response to Axl overexpression under hypoxia conditions, as measured by ELISA (all distributed in two or three independent experiments). Data represent mean ± SEM. **P* < 0.05, *****P* < 0.0001 compared to indicated group, as analyzed by unpaired *t* test or One-way ANOVA test.


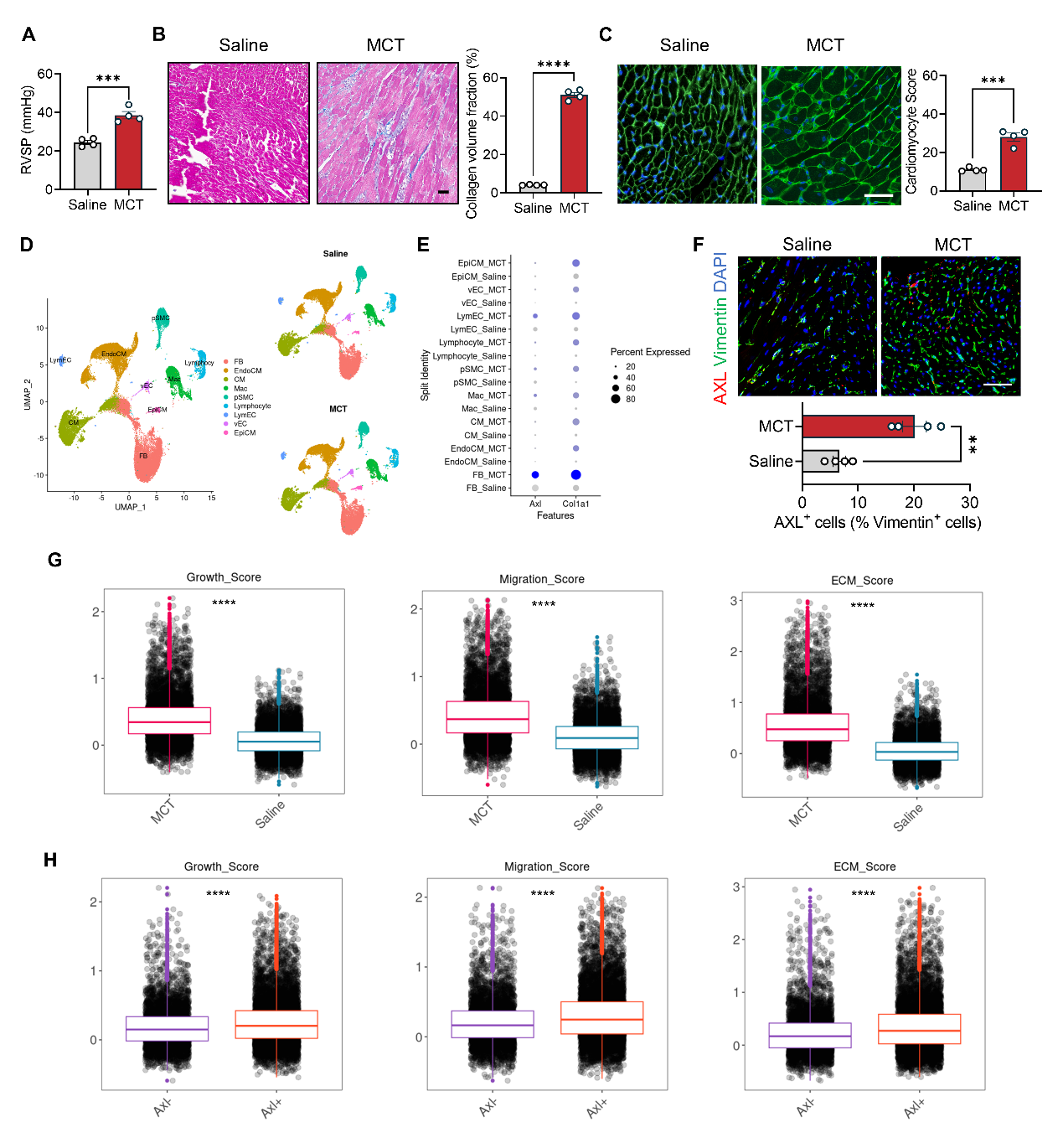


**Figure S17.** **Expression patterns of *Axl* in the RVs from rats administrated with MCT. (A)** Right ventricular systolic pressure (RVSP) was measured by right heart catheterization in rats 4 weeks after MCT or saline administration (n=4/group). **(B)** Representative images and quantification of Masson staining in RVs of MCT-treated or saline-treated rats (n=4/group). **(C)** Representative images and quantification of WGA staining in RVs of MCT-treated or saline-treated rats (n=4/group) **(D)** UMAP visualization of cell clustering across RV tissues of MCT-induced PH rats and saline-treated rats. **(E)** Expression patterns of *Axl* and *Col1a1* across different cell types in RVs of MCT-treated or saline-treated rats. **(F)** Representative images and quantification of immunofluorescent staining for AXL in RVs of MCT-treated or saline-treated rats (n=4/group); **(G)** Growth score, migration scoreand ECM score of RV FBs from MCT-treated or saline-treated rats. Each dot represents an individual FB. **(H)** Growth score, migration score and ECM score of *Axl*-positive or *Axl*-negative FBs in RV tissues. Each dot represents an individual FB. Data represent mean ± SEM. ***P* < 0.01, ****P* < 0.001, *****P* < 0.0001 compared to indicated group, as analyzed by unpaired *t* test or Wilcoxon test. Scale bar = 50 μm.


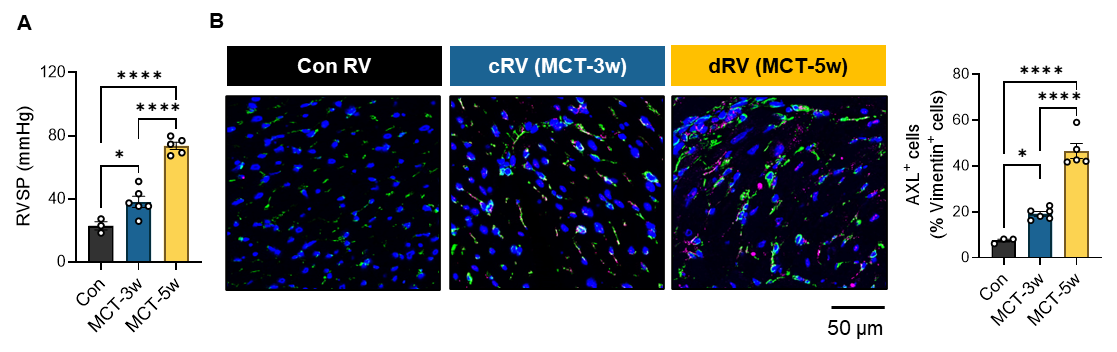


**Figure S18. Temporal profiling of AXL expressions in RVs of MCT-induced PH rats. (A)** Right ventricular systolic pressure in control rats and rats 3-week (compensated state) or 5-week (decompensated state) post MCT administration (n=3-6/group). **(B)** Representative images of double immunofluorescent staining against AXL and Vimentin and quantification of AXL in RV FBs from control rats, rats 3-week (compensated state) or 5-week (decompensated state) post MCT administration (n=3-6/group). Data represent mean ± SEM. * *P* < 0.05, *** *P* < 0.001, *****P* < 0.0001 compared to indicated group, as analyzed by One-way ANOVA test. Scale bar: 50 μm.

**
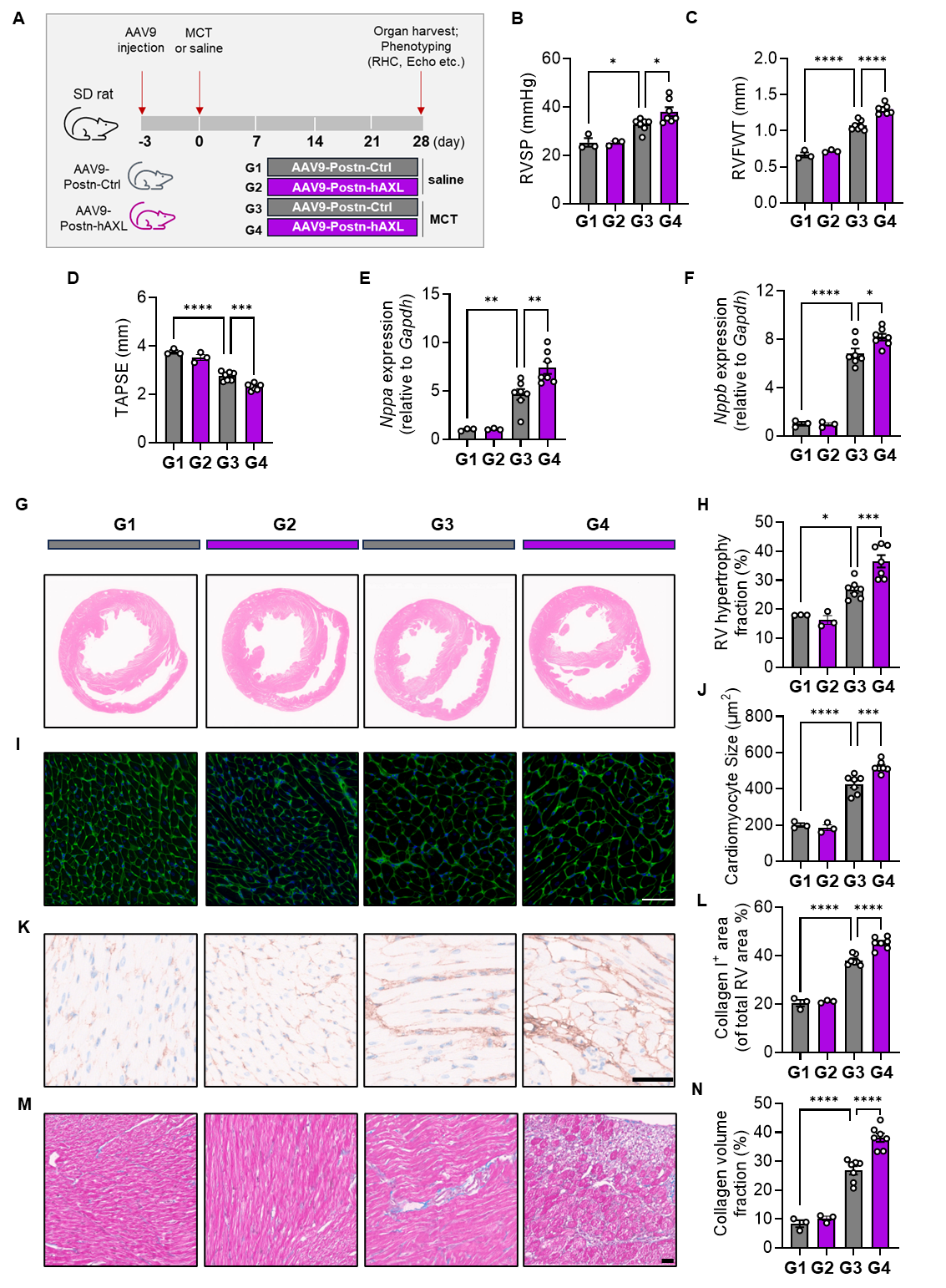
**

**Figure S19.** ***Axl* overexpression in cardiac FBs aggravates RV remodeling in MCT-induced PH.** **(A)** Experimental scheme illustrating the treatment of rats receiving AAV9-Postn-hAXL to overexpress AXL in cardiac FBs or AAV9-Postn-Ctrl, followed by MCT or saline injections. Phenotyping and organ harvest were performed four weeks post MCT or saline injection (G1-G4 groups as indicated). **(B-F)** Right ventricular systolic pressure (RVSP; n=3-7/group) **(B)**; right ventricular free wall thickness (RVFWT; n=3-7/group) **(C)** and tricuspid annular plane systolic excursion (TAPSE; n=3-7/group) **(D)** in rats receiving AAV9-Postn-hAXL or AAV9-Postn-Ctrl with MCT or saline administration. **(E, F)** The transcriptional levels of *Nppa* **(E)** and *Nppb* **(F)** in RV tissues from MCT or control rats receiving AAV9-Postn-hAXL or AAV9-Postn-Ctrl (n=3-7/group). **(G-N)** Representative HE staining **(G)** and assessment **(H)** of right ventricular size measured by right ventricular hypertrophy fraction; WGA staining **(I)** and cardiomyocyte size by cardiomyocyte score **(J)**; IHC staining for collagen I **(K)** and the abundance of collagen deposition **(L)** measured by collagen I-positive area; Masson staining for fibrosis **(M)** and the quantification **(N)** of fibrotic area in RV tissues from MCT or control rats receiving AAV9-Postn-hAXL or AAV9-Postn-Ctrl, respectively. n=3-7/group; Scale bar: 20 μm. Data represent mean ± SEM. * *P* < 0.05, ** *P* < 0.01, *** *P* < 0.001, **** *P* < 0.0001 compared to indicated group, as analyzed by One-way ANOVA test or Kruskal-Wallis test as appropriate.


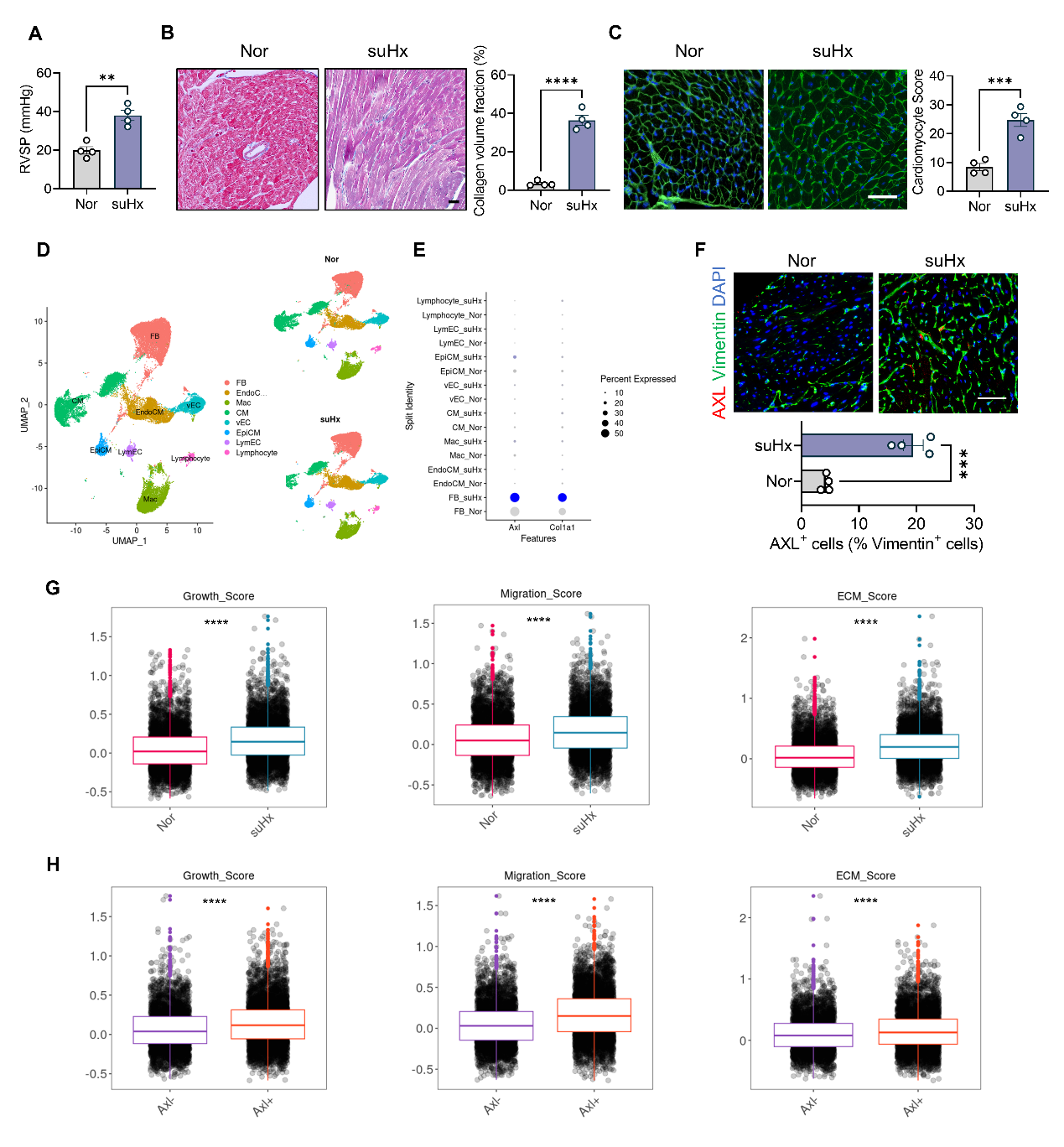


**Figure S20. Expression patterns of *Axl* in the RVs from mice after treatment with SU5416 and hypoxia. (A)** RVSP was measured by right heart catheterization in mice 4 weeks after exposure to SU5416 and hypoxia (suHx) challenge or under normoxia (n=4/group). **(B)** Representative images and quantification of Masson staining in RVs from suHx group or control group (n=4/group). **(C)** Representative images and quantification of WGA staining in RVs from suHx group or control group (n=4/group). **(D)** UMAP visualization of cell clustering across RV tissues from suHx mice and those in normoxic conditions. **(E)** Expression patterns of *Axl* and *Col1a1* across different cell types in RVs from suHx mice or control mice. **(F)** Representative images and quantification of immunofluorescent staining for AXL in RVs from suHx mice or control mice (n=4/group); **(G)** Growth score, migration score and ECM score of RV FBs from suHx mice or control mice. Each dot represents an individual FB. **(H)** Growth score, migration score and ECM score of *Axl*-positive or *Axl*-negative FBs in RVs. Each dot represents an individual FB. Data represent mean ± SEM. ***P* < 0.01, ****P* < 0.001, *****P* < 0.0001 compared to indicated group, as analyzed by unpaired *t* test or Wilcoxon test. Scale bar = 50 μm.


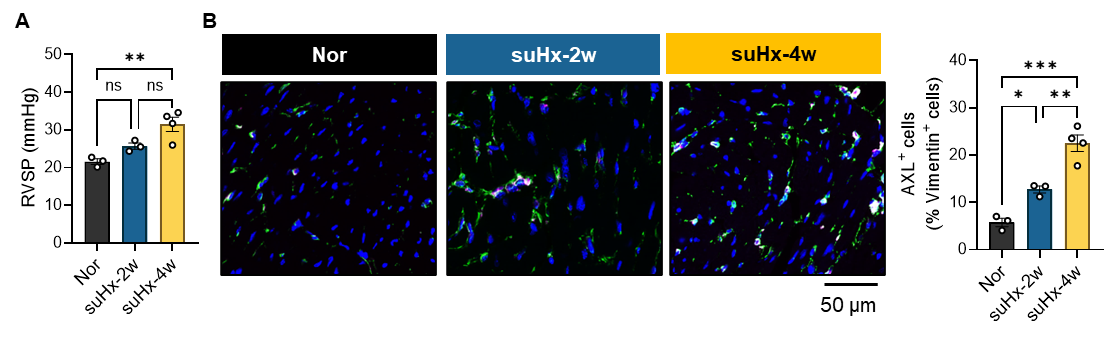


**Figure S21.** **Temporal profiling of AXL expressions in RVs of suHx-induced PH mice.** **(A)** Right ventricular systolic pressure in control mice and mice subjected to 2-week or 4-week suHx exposure (n=3-4/group). **(B)** Representative images of double immunofluorescent staining against AXL and Vimentin and quantification of AXL in RV FBs from control mice, mice subjected to 2-week or 4-week suHx exposure (n=3-4/group). Data represent mean ± SEM. * *P* < 0.05, *** *P* < 0.001, *****P* < 0.0001 compared to indicated group, as analyzed by One-way ANOVA test. Scale bar: 50 μm.


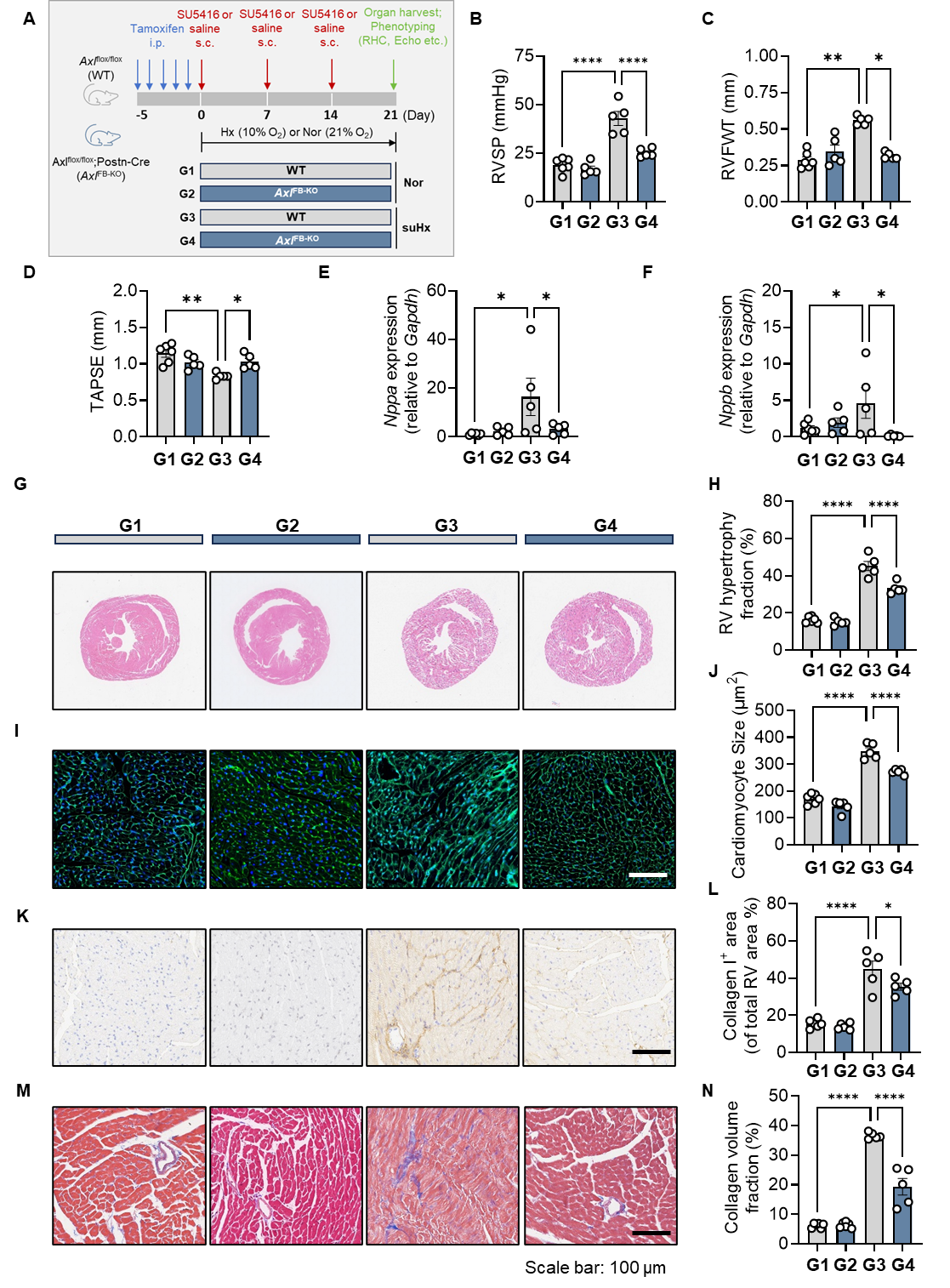


**Figure S22. Genetic ablation of Axl in cardiac FBs attenuated RV remodeling after suHx challenge. (A)** Experimental scheme showing that *Axl*^flox/flox^; PostnCre mice (genetic ablation of Axl in cardiac FBs; *Axl*^FB-KO^) mice and *Axl*^flox/flox^ mice received three weekly subcutaneous injections of Sugen 5416 (one dose per week) combined with concurrent hypoxia exposure for three weeks, or saline injections under ambient air conditions (G1-G4 groups as indicated). **(B-D)** Right ventricular systolic pressure (RVSP; n=5-6/group) **(B)**; right ventricular free wall thickness (RVFWT; n=5-6/group) **(C)**; and tricuspid annular plane systolic excursion (TAPSE; n=5-6/group) **(D)** in *Axl*^FB-KO^ or *Axl*^flox/flox^ mice following suHx exposure or in ambient. **(E, F)** The transcriptional levels of *Nppa* **(E)** and *Nppb* **(F)** in RV tissues from *Axl*^FB-KO^ or *Axl*^flox/flox^ mice following suHx challenge or in ambient (n=5-6/group). **(G-N)** Representative HE staining **(G)** and assessment **(H)** of right ventricular size measured by right ventricular hypertrophy fraction; WGA staining **(I)** and cardiomyocyte size **(J)**; IHC staining for collagen I **(K)** and the abundance of collagen deposition **(L)** measured by collagen I-positive area; Masson staining for fibrosis **(M)** and the quantification **(N)** of fibrotic area in RV tissues from *Axl*^FB-KO^ or *Axl*^flox/flox^ mice following suHx challenge or in ambient, respectively. n=5-6/group. Data represent mean ± SEM. * *P* < 0.05, ** *P* < 0.01, *** *P* < 0.001, **** *P* < 0.0001 compared to indicated group, as analyzed by One-way ANOVA test or Kruskal-Wallis test as appropriate.


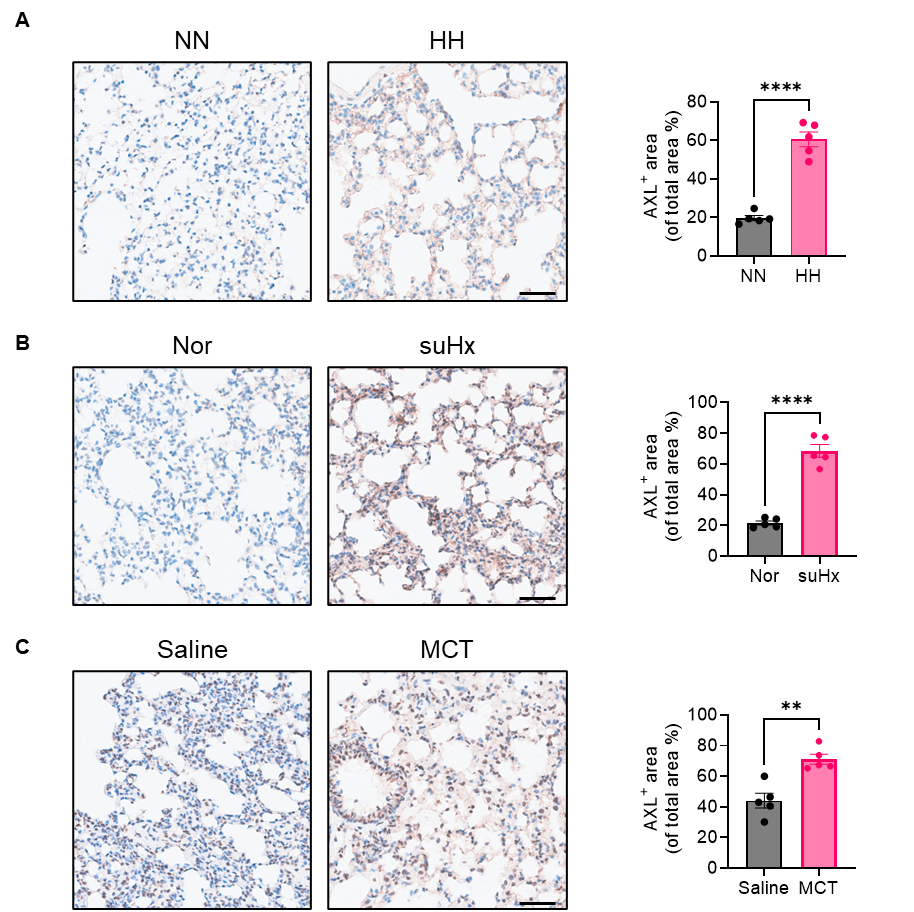


**Figure S23.** **AXL protein levels in the lungs of PH animal models.** **(A)** Representative immunohistochemistry (IHC) images and quantitative analysis of Axl expression in lung tissues from HH-induced PH and control mice (n=5/group). **(B)** Representative IHC images and quantification of Axl expression in lung tissues from suHx-induced PH and control mice (n=5/group). **(C)** Representative IHC images and quantification of Axl expression in lung tissues from MCT-induced PH and control rats (n=5/group). Data are represented as mean ± SEM. ** *P* < 0.01, *****P* < 0.0001 compared to control group, as analyzed by unpaired *t* test. Scale bar: 50 μm.


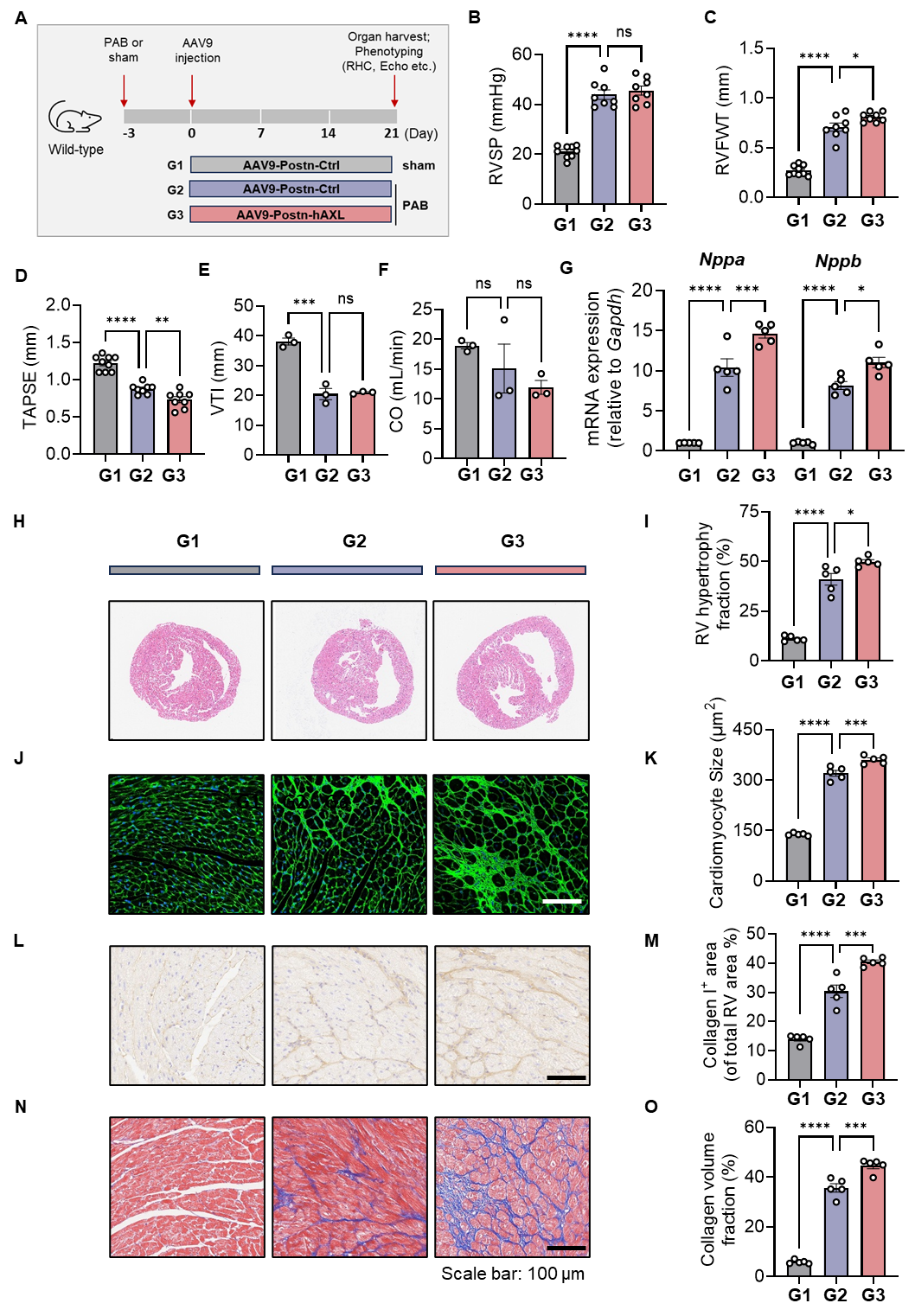


**Figure S24. *Axl* overexpression in cardiac FBs aggravates RV remodeling in PAB mouse model.** **(A)** Experimental scheme illustrating the treatment of mice with AAV9-Postn-hAXL (to overexpress AXL in cardiac FBs) or AAV9-Postn-Ctrl, followed by subjection to PAB or sham surgery. Phenotyping and organ harvest were performed three weeks post-surgery (G1-G3 groups as indicated). **(B-D)** Right ventricular systolic pressure (RVSP; n=8-9/group) **(B)**; right ventricular free wall thickness (RVFWT; n=8-9/group) **(C)**; tricuspid annular plane systolic excursion (TAPSE; n=8-9/group) **(D)**; velocity times integral (VTI; n=3/group) **(E)** and cardiac output (CO; n= 3/group) **(F)** in mice receiving AAV9-Postn-hAXL or AAV9-Postn-Ctrl followed by PAB or sham procedure. **(G)** The transcriptional levels of *Nppa* and *Nppb* in RV tissues from PAB or sham mice receiving AAV9-Postn-hAXL or AAV9-Postn-Ctrl (n=5-6/group). **(H-O)** Representative HE staining **(H)** and assessment **(I)** of right ventricular size measured by right ventricular hypertrophy fraction; WGA staining **(J)** and cardiomyocyte size **(K)**; IHC staining for collagen I **(L)** and the abundance of collagen deposition **(M)** measured by collagen I-positive area; Masson staining for fibrosis **(N)** and the quantification **(O)** of fibrotic area in RV tissues from PAB or sham mice receiving AAV9-Postn-hAXL or AAV9-Postn-Ctrl, respectively. n=5-6/group. Data represent mean ± SEM. * *P* < 0.05, ** *P* < 0.01, *** *P* < 0.001, **** *P* < 0.0001 compared to indicated group, as analyzed by One-way ANOVA test or Kruskal-Wallis test as appropriate.


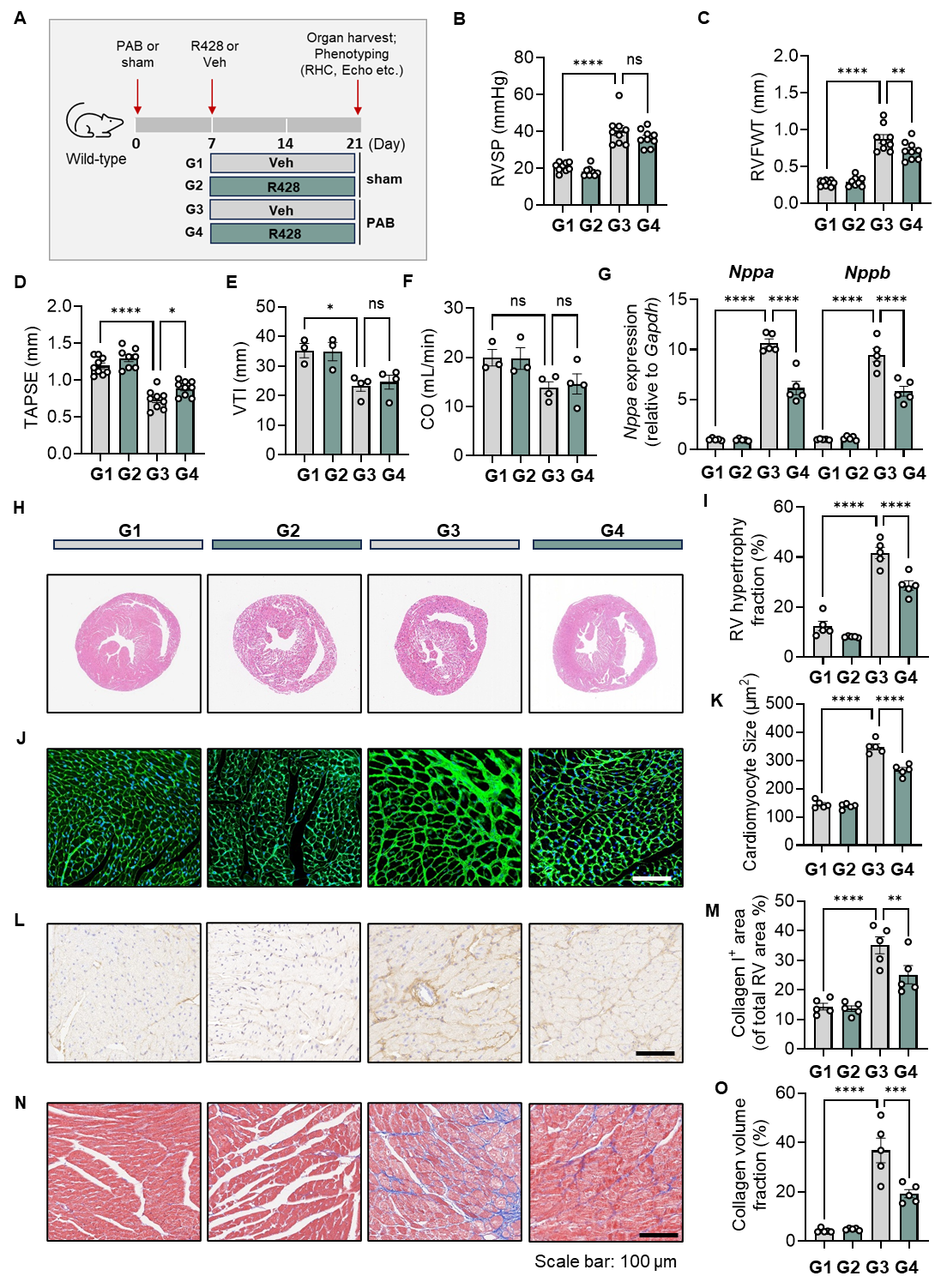


**Figure S25. R428 treatment rescues RV remodeling in PAB mouse model.** **(A)** Experimental scheme showing that mice were subjected to PAB or sham surgery and subsequently administered R428 or vehicle one week later (G1-G4 groups as indicated). **(B-D)** Right ventricular systolic pressure (RVSP; n=8-9/group) **(B)**; right ventricular free wall thickness (RVFWT; n=8-9/group) **(C)**; tricuspid annular plane systolic excursion (TAPSE; n=8-9/group) **(D)**; velocity times integral (VTI; n=3-4/group) **(E)** and cardiac output (CO; n= 3-4/group) **(F)** in mice that underwent PAB or sham surgery and received treatment with R428 or vehicle. **(G)** The transcriptional levels of *Nppa* and *Nppb* in RV tissues from PAB or sham mice receiving R428 or vehicle administration (n=5-6/group). **(H-O)** Representative HE staining **(H)** and assessment **(I)** of right ventricular size measured by right ventricular hypertrophy fraction; WGA staining **(J)** and cardiomyocyte size **(K)**; IHC staining for collagen I **(L)** and the abundance of collagen deposition **(M)** measured by collagen I-positive area; Masson staining for fibrosis **(N)** and the quantification **(O)** of fibrotic area in RV tissues from PAB or sham mice receiving R428 or vehicle treatment, respectively. n=5-6/group. Data represent mean ± SEM. * *P* < 0.05, ** *P* < 0.01, *** *P* < 0.001, **** *P* < 0.0001 compared to indicated group, as analyzed by One-way ANOVA test or Kruskal-Wallis test as appropriate.


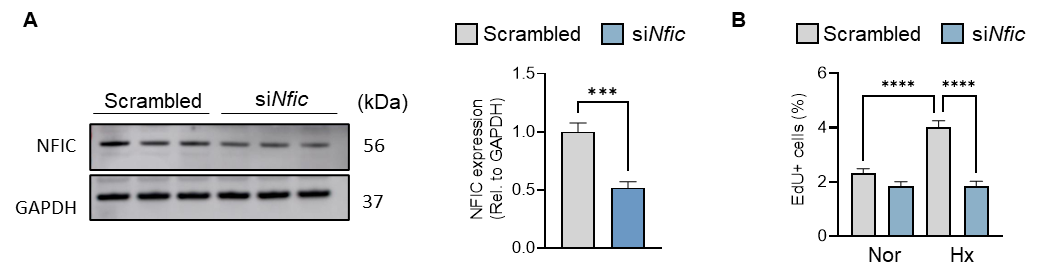


**Figure S26. The effect of *Nfic* knockdown on the proliferation of FBs in response to hypoxia. (A)** NFIC protein level was reduced in si*Nfic*-transfected primary cardiac FBs compared to scrambled siRNA transfected cells (all distributed in two experiments). **(B)** The proliferation rate of primary mouse FBs transfected with si*Nfic* (20 nM) or scrambled siRNA (20 nM) in response to hypoxia for 24 h, as measured by EdU incorporation assay (distributed in four independent experiments). Data represent mean ± SEM. ****P* < 0.001 or *****P* < 0.0001 compared to indicated group, as analyzed by unpaired *t* test or One-way ANOVA test.


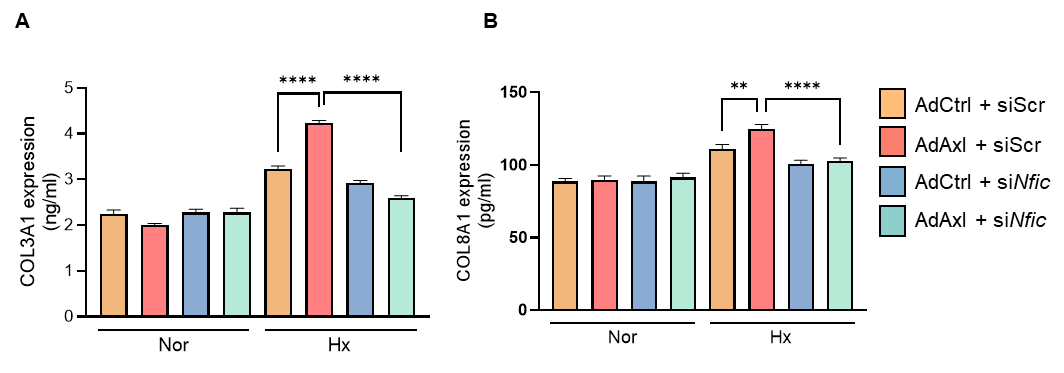


**Figure S27. The effect of *Nfic* knockdown on synthesis of collagen in FBs infected with AdAxl or AdCtrl under hypoxic conditions.** **(A-B)** *Nfic* silencing reduced the protein levels of COL3A1 **(A)** and COL8A1 **(B)** in cell lysates from primary mouse FBs in response to Axl overexpression under hypoxic conditions, as measured by ELISA (all distributed in three independent experiments). Data represent mean ± SEM. ***P* < 0.01, *****P* < 0.0001 compared to indicated group, as analyzed by One-way ANOVA test.


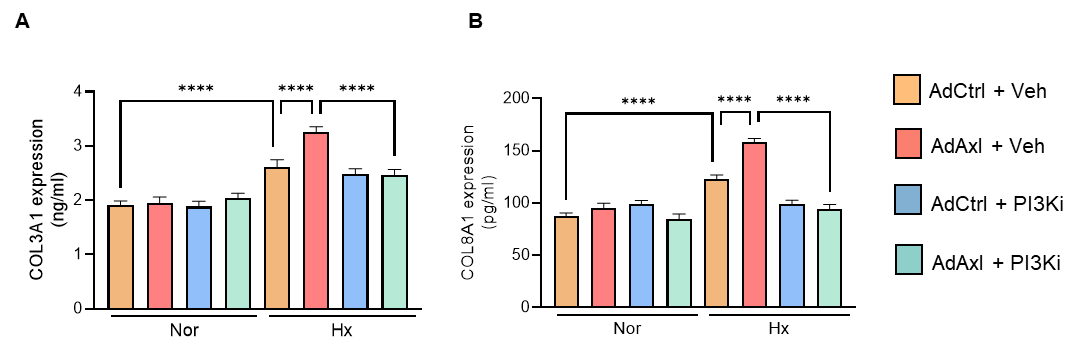


**Figure S28.** **The effect of PI3K inhibitor on synthesis of collagen in FBs infected with AdAxl or AdCtrl under hypoxic conditions.** **(A-B)** PI3K inhibitor reduced the protein levels of COL3A1 **(A)** and COL8A1 **(B)** in cell lysates from primary mouse FBs in response to Axl overexpression under hypoxic conditions, as measured by ELISA (all distributed in three independent experiments). Data represent mean ± SEM. *****P* < 0.0001 compared to indicated group, as analyzed by One-way ANOVA test.


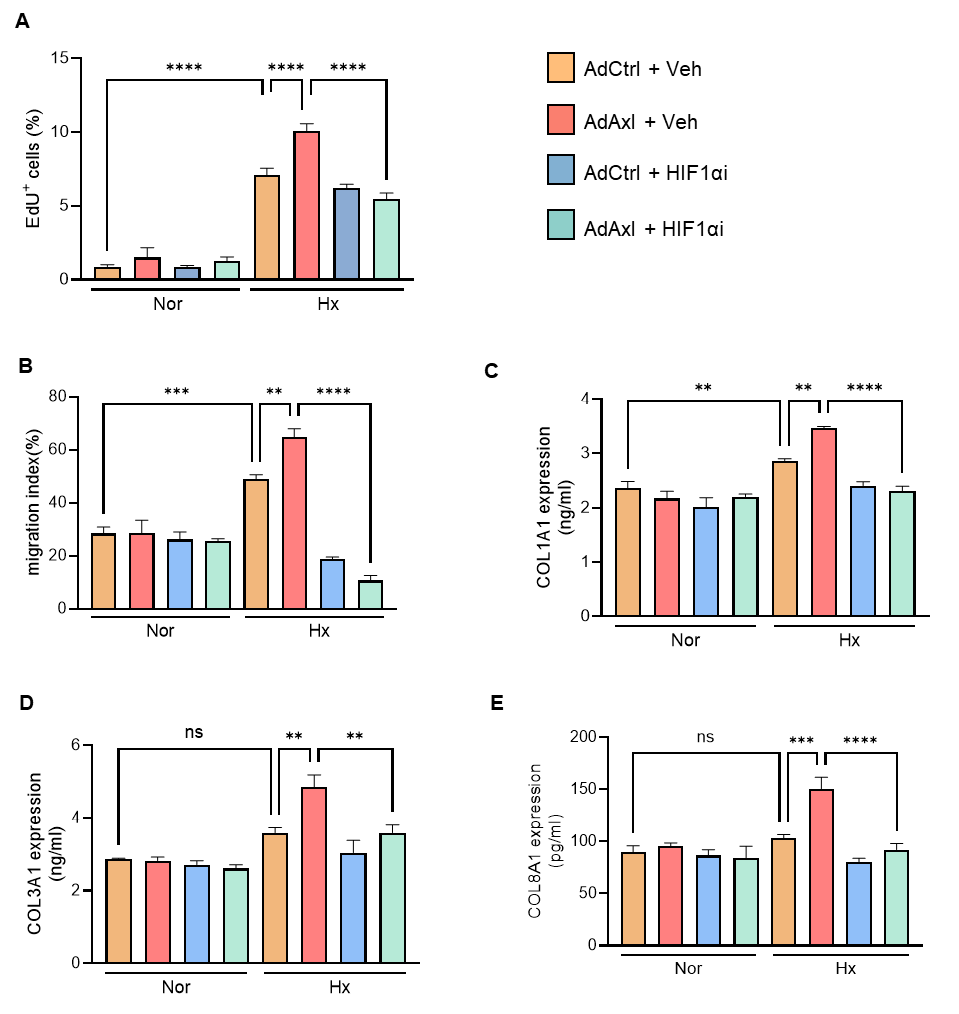


**Figure S29. The effect of HIF1α inhibitor on phenotypes of FBs infected with AdAxl or AdCtrl under hypoxic conditions. (A)** The proliferation rate of primary mouse FBs treated with HIF1α inhibitor or vehicles in response to hypoxia for 24 h, as measured by EdU incorporation assay (distributed in two independent experiments). **(B)** The migration rate of primary mouse FBs treated with HIF1α inhibitor or vehicles in response to hypoxia for 24 h, as measured by scratch assay (distributed in two independent experiments). **(C-E)** HIF1α inhibitor reduced the protein levels of COL1A1 **(C)**, COL3A1 **(D)** and COL8A1 **(E)** in cell lysates from primary mouse FBs in response to Axl overexpression under hypoxic conditions, as measured by ELISA (all distributed in two independent experiments). Data represent mean ± SEM. ***P* < 0.01, ****P* < 0.001, *****P* < 0.0001 compared to indicated group, as analyzed by One-way ANOVA test; ns indicates not significant.


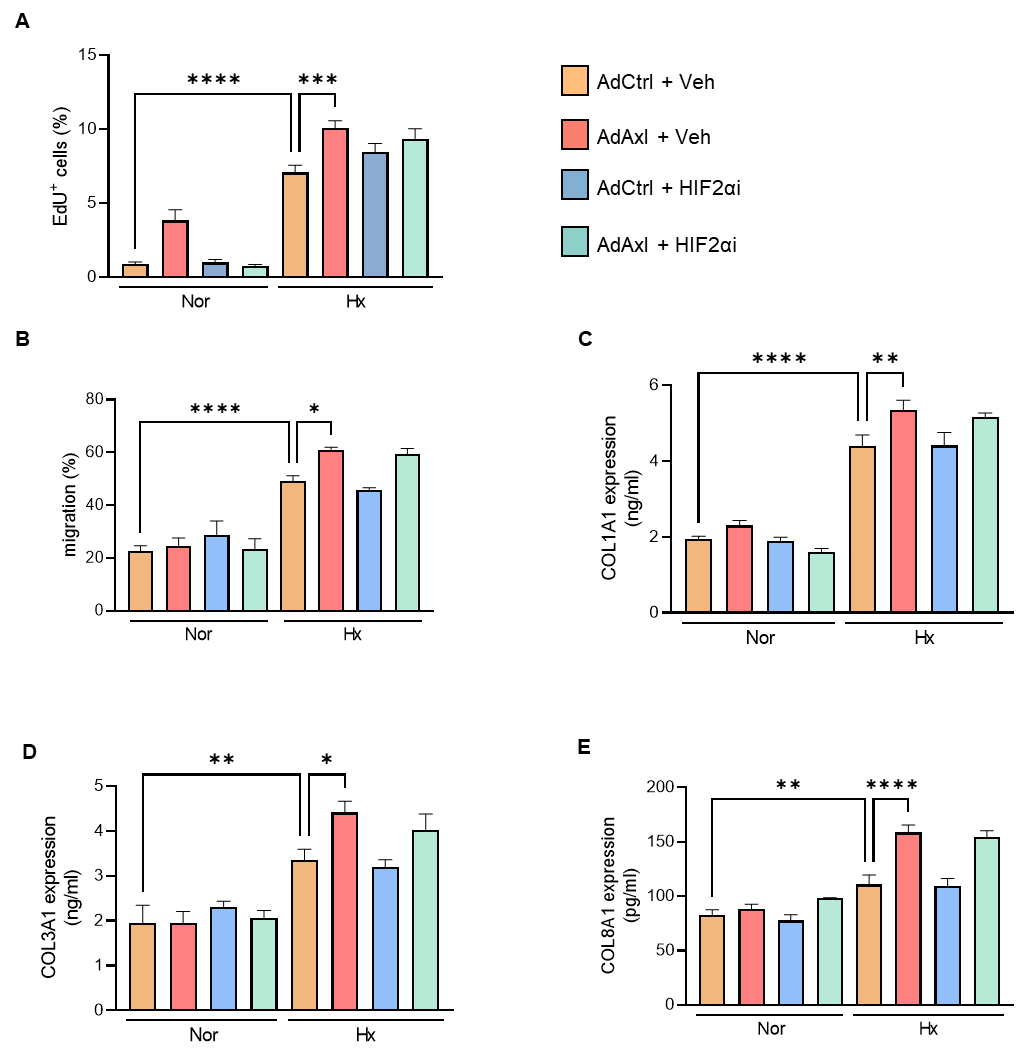


**Figure S30. The effect of HIF2α inhibitor on phenotypes of FBs infected with AdAxl or AdCtrl under hypoxic conditions.** **(A)** The proliferation rate of primary mouse FBs treated with HIF2α inhibitor or vehicles in response to hypoxia for 24 h, as measured by EdU incorporation assay (distributed in two independent experiments). **(B)** The migration rate of primary mouse FBs treated with HIF2α inhibitor or vehicles in response to hypoxia for 24 h, as measured by scratch assay (distributed in two independent experiments). **(C-E)** HIF2α inhibitor didn’t rescue the increased protein levels of COL1A1 **(C)**, COL3A1 **(D)** and COL8A1 **(E)** in cell lysates from primary mouse FBs in response to Axl overexpression under hypoxic conditions, as measured by ELISA (all distributed in two independent experiments). Data represent mean ± SEM. **P* < 0.05, ***P* < 0.01, ****P* < 0.001, *****P* < 0.0001 compared to indicated group, as analyzed by One-way ANOVA test.
